# Unique structural features define the decarboxylation activity of a CYP152 fatty acid decarboxylase from *Lacicoccus alkaliphilus*

**DOI:** 10.1016/j.jbc.2025.110397

**Published:** 2025-06-19

**Authors:** Suppalak Phaisan, Aisaraphon Phintha, Duangthip Trisrivirat, Narin Lawan, Jeerus Sucharitakul, Ailada Charoenpol, Pratchaya Watthaisong, Hideaki Tanaka, Genji Kurisu, Pimchai Chaiyen

**Affiliations:** 1 School of Biomolecular Science and Engineering, Vidyasirimedhi Institute of Science and Technology (VISTEC), Rayong, Thailand; 2 Faculty of Science, Department of Chemistry, Chiang Mai University, Chiang Mai, Thailand; 3 Faculty of Dentistry, Department of Biochemistry, Chulalongkorn University, Bangkok, Thailand; 4 Techno Pro, Inc., TechnoPro R&D Company, Kobe, Hyogo Japan; 5 Institute for Protein Research, Osaka University, Osaka, Japan; 6 Department of Macromolecular Science, Osaka University, Osaka, Japan

**Keywords:** cytochrome P450, CYP152, fatty acid decarboxylation, fatty acid hydroxylation, alkene formation, decarboxylase, hydroxylase, peroxygenase, reductase

## Abstract

Cytochrome P450 CYP152s catalyze decarboxylation of fatty acids to generate terminal alkenes, valuable compounds for various industries. Here, we identified, overexpressed, and characterized a new CYP152 enzyme from *Lacicoccus alkaliphilus* (OleT_LA_) and compared its biophysical and biochemical properties with the well-studied OleT_JE_ from *Jeotgalicoccus* sp. 8456. Improved expression protocols gave the highest yields of CYP152 holoenzymes reported to date. OleT_LA_ exhibits twice the catalytic turnover number of OleT_JE_ when using hexadecanoic acid and H_2_O_2_ as substrates in 10% (v/v) ethanol (EtOH). The X-ray structure of OleT_LA_ in complex with icosanoic acid revealed a unique flipped heme and a substrate tunnel configuration which are different than those of other CYP152 decarboxylases. Molecular dynamics simulations revealed that in the presence of EtOH, OleT_LA_ displays structural dynamics which maintain structural interactions better than those of OleT_JE_. As I178 in OleT_LA_ (equivalent to L176 in OleT_JE_) shows close interactions with its substrate during simulations, I178L of OleT_LA_ and L176I of OleT_JE_ variants were constructed and investigated for their activities. While L176I in OleT_JE_ caused a significant loss of activity, I178L of OleT_LA_ had activities that were equivalent to or greater than those of the wild-type enzyme, suggesting that overall scaffold of OleT_LA_ is more amenable to mutation than OleT_JE_. Stopped-flow investigations of OleT_LA_ reactions indicated that EtOH increases the rate constant of Compound I formation. We also identified a new redox partner system, ferredoxin and ferredoxin reductase that can function as effective electron donors for both *in vitro* and *in vivo* systems of CYP152s.

Terminal alkenes are important building blocks for industries as they are used for synthesis of lubricants, surfactants, biofuels and polymer materials. Olefins are typically synthesized from petroleum-based feedstocks using steam cracking processes in which their production processes require high temperatures and pressures and are not green for the environment (1, 2). Enzymatic reactions to synthesize olefins are thus attractive for the sustainable production of olefins for future bio-industries.

Several types of fatty acid decarboxylases, including non-heme mononuclear iron-dependent decarboxylases (UndA and UndB) and cytochrome P450-dependent enzymes in the CYP152 family, can catalyze the bioconversion of medium- and long-chain fatty acids to generate aliphatic olefins. UndA and UndB prefer to use medium-chain fatty acids of C10 to C14 (3) and C10 to C16 lengths, respectively (4, 5, 6), while CYP152 enzymes prefer to use medium to long-chain (C10 to C20) fatty acids (5, 7). As medium to long-chain fatty acids are abundant in food and agro-industrial waste such as cooking oils, animal fat, and plant oil mill effluent, the reactions of CYP152 enzymes are thus attractive for bio-based, circular, and sustainable economy models for producing valuable chemicals from bio-based waste (8).

The CYP152 enzymes typically exhibit mixed activities between fatty acid decarboxylase and hydroxylase. CYP152 fatty acid hydroxylases currently known to date include CYP152A1 (9) and CYP152T1 (10), which produce β-hydroxy fatty acids as major products while CYP152A2 (11), CYP152A8 (12), CYP152B1 (13), CYP152K6 (14), CYP152N1 (15), and P450_Jα_ (16) produce α-hydroxy fatty acids as major products. For CYP152_MP_ (17), γ, δ, ε-hydroxy fatty acids were detected along with β-hydroxy fatty acids. With regard to decarboxylation activities, CYP152L1 (OleT_JE_) (10, 18, 19), CYP152L2 (12), CYP152L7, CYP152L8, CYP152_JH_ (20), CYP152_MC_ (21), CYP152T7, CYP152T8 (10), and OleTP_RN_ (22) have been shown to produce terminal alkenes as their major products. Although several CYP152 decarboxylases have been identified and investigated, OleT_JE_ from *Jeotgalicoccus* sp. 8456 remains the most extensively investigated biocatalyst of this class. OleT_JE_ exhibits the highest catalytic efficiency and has the best chemoselectivity for decarboxylation among all known CYP152 enzymes (*k*_cat_ = 71 min^−1^, and *k*_cat_/*K*_M_ of tetradecanoic acid (C14FA) decarboxylation = 2.9 μM^−1^ min^−1^) (18).

Although the properties of OleT_JE_ are attractive for biocatalysis, improvements are still needed for its practical application. The enzyme can bypass the use of redox partners to supply electrons by efficiently using H_2_O_2_ as a co-substrate *via* a peroxide shunt pathway, enabling a convenient *in vitro* bioconversion process. However, the use of the H_2_O_2_ shunt is not suitable for whole cell biocatalysis because H_2_O_2_ at concentrations higher than 2 mM is harmful to the cell (23). Previous reports have shown that OleT_JE_ can use various types of redox partners (18) to drive the catalytic cycle such as using Putidaredoxin reductase (CamA) and Putidaredoxin (CamB). The use of CamA and CamB was shown to provide higher yields of short chain alkenes than the H_2_O_2_ system (24). Despite various studies on *in vitro* bioconversion, reports on using CYP152 and its redox partners for whole cell biocatalysis are still limited (21, 25). Although OleT_JE_ can use a wide range of fatty acids as substrates, their product yields are often limited by substrate solubility (12, 18, 20). Addition of a co-solvent has been shown to increase the solubility of hydrophobic substrates and thus product yields (26, 27, 28, 29). However, the process would require the biocatalyst employed to be solvent tolerant. The impact of co-solvents on the bioconversion of CYP152 enzymes remains largely unexplored. Only one study has shown that using 5% (v/v) acetone can enhance the total turnover number (TTN) of OleT_JE_ decarboxylation of dodecanoic acid (C12FA), hexadecanoic acid (C16FA), and octadecanoic acid (C18FA). However, the exact mechanism behind this increase has not been thoroughly investigated.

CYP152 decarboxylases including OleT_JE_ generally are not overexpressed well in soluble holoenzyme forms. Previous studies mostly obtained yields of 1.5 to 25 mg of purified CYP152s from 1 L of culture medium (14, 15, 19), with the highest expression yield being reported for purified P450_Jα_ at 49 mg/1 L of culture medium (16). Moreover, it was reported that purified OleT_JE_ variants have shown only around 25% to 35% of heme incorporation (19). Therefore, improvement in holoenzyme expression is needed for future practical applications as well as for performing mechanistic studies of CYP152 decarboxylases.

In this work (Fig. 1), we have identified, overexpressed, and investigated the catalytic, biochemical, and biophysical properties of a new CYP152 from *Lacicoccus alkaliphilus* (OleT_LA_) and compared these properties to those of OleT_JE_. The overexpression system has been improved to increase yields of soluble OleT_LA_ and OleT_JE_ holoenzymes. We successfully solved the X-ray structure of OleT_LA_ in complex with icosanoic acid (C20FA), investigated substrate scope, and performed extensive steady-state kinetics analyses with hexadecanoic acid (C16FA) in the presence and absence of EtOH. As the presence of 10% (v/v) of EtOH enhances the decarboxylation activities of OleT_LA,_ we carried out molecular dynamics (MD) simulations to investigate the dynamics of OleT_LA_ in the presence of EtOH and with a substrate. I178L of OleT_LA_ and L176I of OleT_JE_ variants were constructed and investigated for their activities to evaluate the importance of interactions between this residue and the substrate. To further understand the role of EtOH in enhancing OleT_LA_ activities, isothermal titration calorimetry (ITC) characterization of ligand binding and stopped-flow experiments to measure rates of Compound I formation were carried out. Finally, we identified new redox partner systems suitable for both *in vitro* and *in vivo* bioconversion by CYP152s.

**Figure 1:**
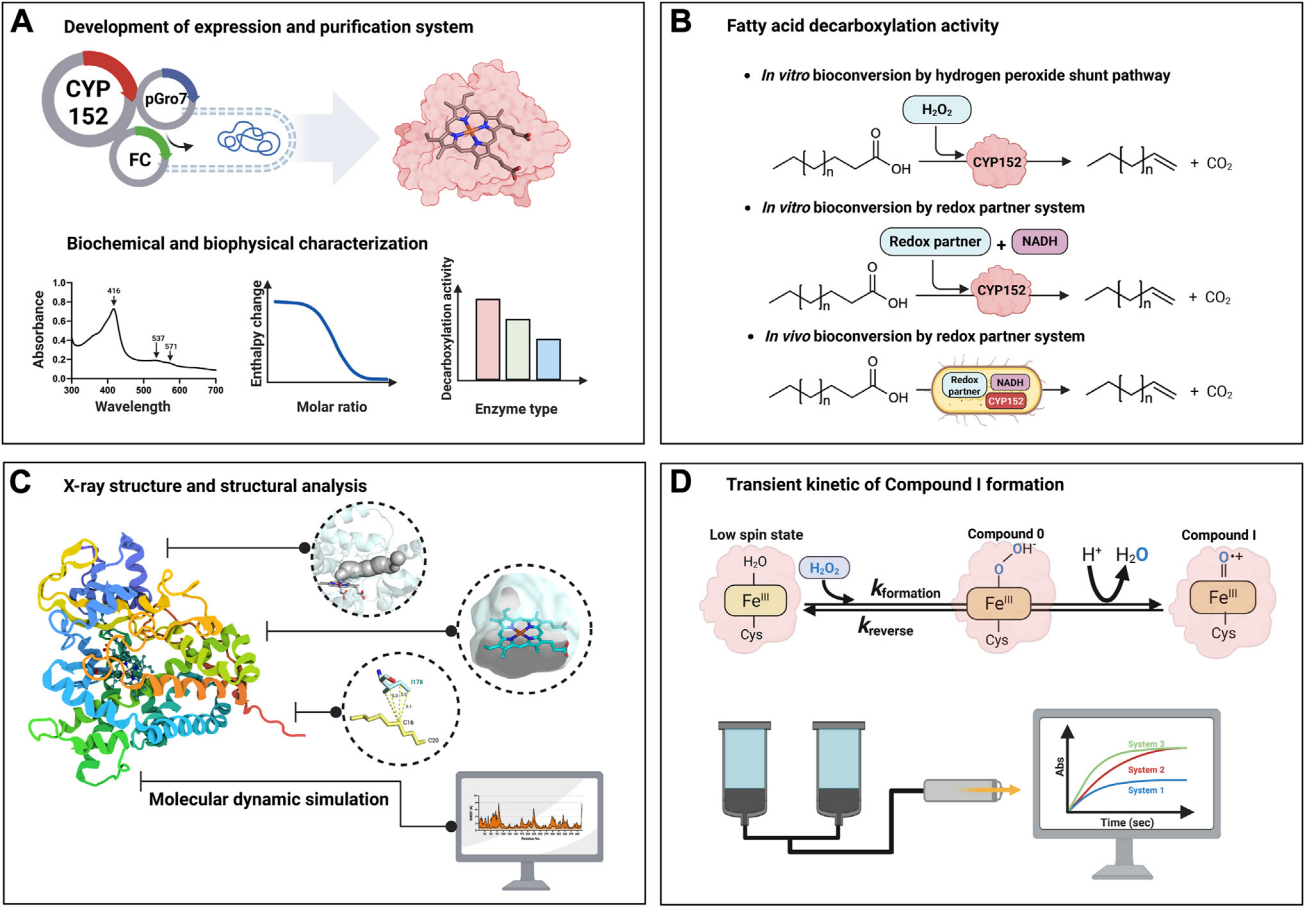
**Overview of investigations in this study.***A,* development of overexpression and purification methods to achieve a high yield of CYP152 holoenzymes from *Lacicoccus alkaliphilus* (OleT_LA_) and from *Jeotgalicoccus* sp. 8456 (OleT_JE_) and investigation of the biochemical and biophysical properties of OleT_LA_. *B,* demonstration of the use of OleT_LA_ for *in vitro* bioconversion *via* the hydrogen peroxide shunt pathway and a new redox partner system, as well as for *in vivo* bioconversion using the new redox partner system. *C,* determination of the X-ray structure of OleT_LA_ in complex with icosanoic acid (C20FA). Structural analysis and molecular dynamics simulations to identify structural features controlling uniqueness of OleT_LA_. *D,* transient kinetics to investigate Compound I formation in the reactions of OleT_LA_ in the presence and absence of ethanol.

## Results

### Enzyme mining and identification of OleT_LA_

To discover a new CYP152 decarboxylase, the Enzyme Function Initiative-Enzyme Similarity Tool (EFI-EST) was used to create a sequence similarity network (SSN) for enzyme mining. Because OleT_JE_ is a benchmark enzyme, we used the OleT_JE_ sequence as a query sequence. OleT_JE_ homologs with sequence identity higher than 60% were searched and retrieved from the UniProt database using the Basic Local Alignment Search Tool (BLAST). The EFI analysis results were used to generate the SSN clusters (Fig. S1). We were interested in CYP152 from *L. alkaliphilus* (OleT_LA_), which is an alkaliphile and moderate halophilic bacterium located in the same cluster as OleT_JE_. Because OleT_LA_ has 75% sequence identity to OleT_JE_, we hypothesized that OleT_LA_ may exhibit distinct characteristics compared to OleT_JE_ but have similarities in thermotolerance and alkaline tolerance characteristics.

**Figure 2:**
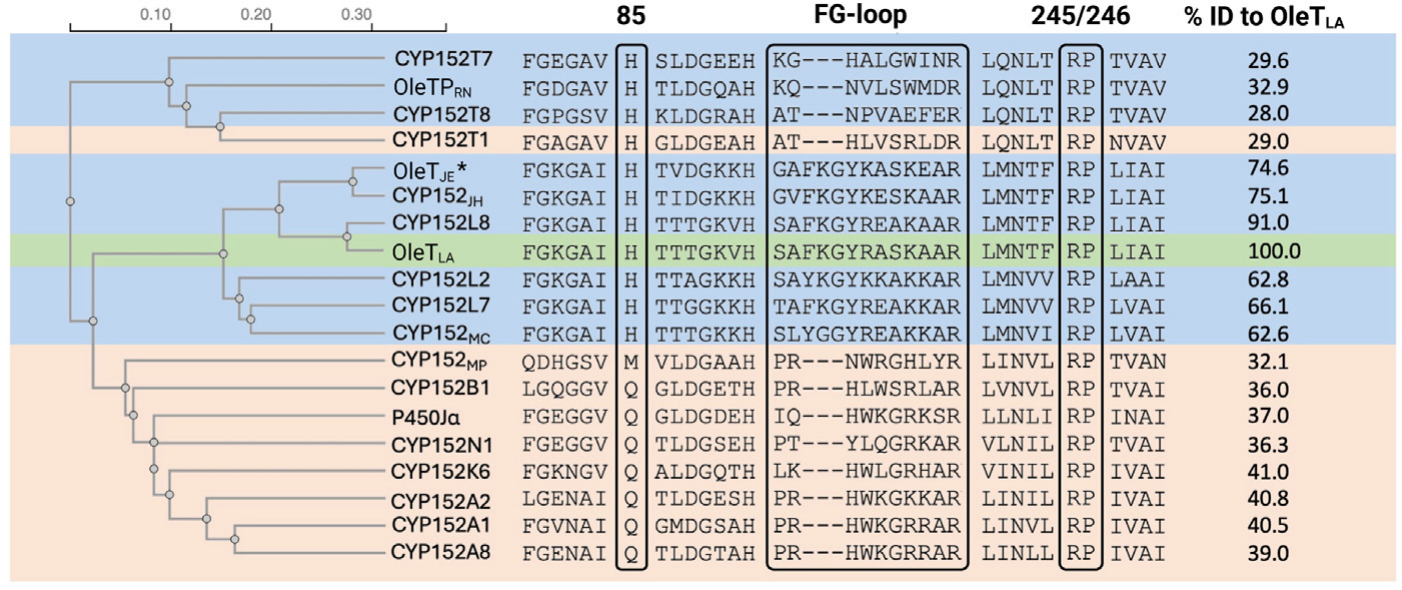
**A phylogenetic tree and multiple sequence alignments of known CYP152s and OleT_LA_**. *Black boxes* indicate conserved positions of the residues 85, FG-loop region, R245 and P246 of OleT_JE_. The last column indicates the percentage identity of each enzyme compared to OleT_LA_. The *blue and orange highlights* show major enzyme activities of decarboxylation and hydroxylation, respectively. The *green highlight* is OleT_LA_. The *black asterisk* is OleT_JE_ which is a query sequence for enzyme mining using ESI-EST.

We further analyzed the sequence of OleT_LA_ compared to other reported CYP152 enzymes. The phylogenetic tree showed that OleT_LA_ is located in the same clade with other CYP152 decarboxylases including OleT_JE_ and CYP152_JH_ and has the relationship closest to CYP152L8 which is a fatty acid decarboxylase (20) (Fig. 2). The identity metrics of OleT_LA_ and other CYP152s are shown in Figure S2 of the supporting information. Multiple sequence alignments of OleT_LA_ with previously reported CYP152 sequences were further used to identify the conserved and crucial residues of the enzyme (Fig. 2). The analysis revealed the presence of the conserved R245, P246, H85, and the FG-loop which were reported to be crucial for the decarboxylation activity of OleT_JE_ (18, 19, 30). Altogether, the bioinformatic analysis suggests that OleT_LA_ should exhibit decarboxylation as its major activity similar to OleT_JE_. We thus selected OleT_LA_ for further studies.

### Improvement in holoenzyme expression and purification

As the production of active holoenzymes is important for obtaining sufficient enzyme amounts for investigations in mechanistic studies and structural characterizations, we optimized expression and purification systems to increase the production of holoenzymes. Although several systems have been shown to increase holoenzyme expression for various P450 enzymes, their effects are not universal and cannot be assumed to be the same for all P450 enzymes. Co-expression of genes related to heme synthesis can enhance holoenzyme expression. Examples of these include the co-overexpression of bacterial P450 from *Streptomyces turgidiscabies* with ferrochelatase (31) or the co-expression of the heme nitric oxide/oxygen-binding (H-NOX) domain with the heme receptor ChuA (ChuA) (32). For P450 27C1, P450 2U1, and P450 2W1, soluble enzyme yield can be increased by co-overexpression with chaperones (33, 34). Although the addition of δ-aminolevulinic acid (δ-ALA) to the culture medium can enhance expression of various P450 enzymes (12, 16, 17, 20, 25, 35, 36), it does not enhance overexpression of OleT_JE_ variants (R245E and R245L) (19).

To the best of our knowledge, we could not find a study that investigated the combined effects of co-expression of ferrochelatase and pGro7, along with the addition of δ-ALA and hemin, on improving the yield of holoenzymes in the P450 enzymes, including the CYP152 family. For the CYP152 enzymes, although supplementation with δ-ALA (12, 16, 17, 19, 20, 25, 36) and co-expression with a chaperone and ChuA (30, 37) have been used for expression, a comprehensive comparison has not been reported. Moreover, most studies used Terrific Broth (TB) and Luria Bertani (LB) with IPTG induction as the culture system, while the use of ZY auto-induction medium (which uses lactose rather than the expensive IPTG) has not yet been explored for CYP152s. We were interested in investigating the use of ZY to overexpress CYP152 enzymes because ZY auto-induction medium has been reported to yield higher expression of target proteins under the control of the T7-lac promoter and allow for culture growth to higher cell densities than TB or LB medium (38, 39).

**Figure 3:**
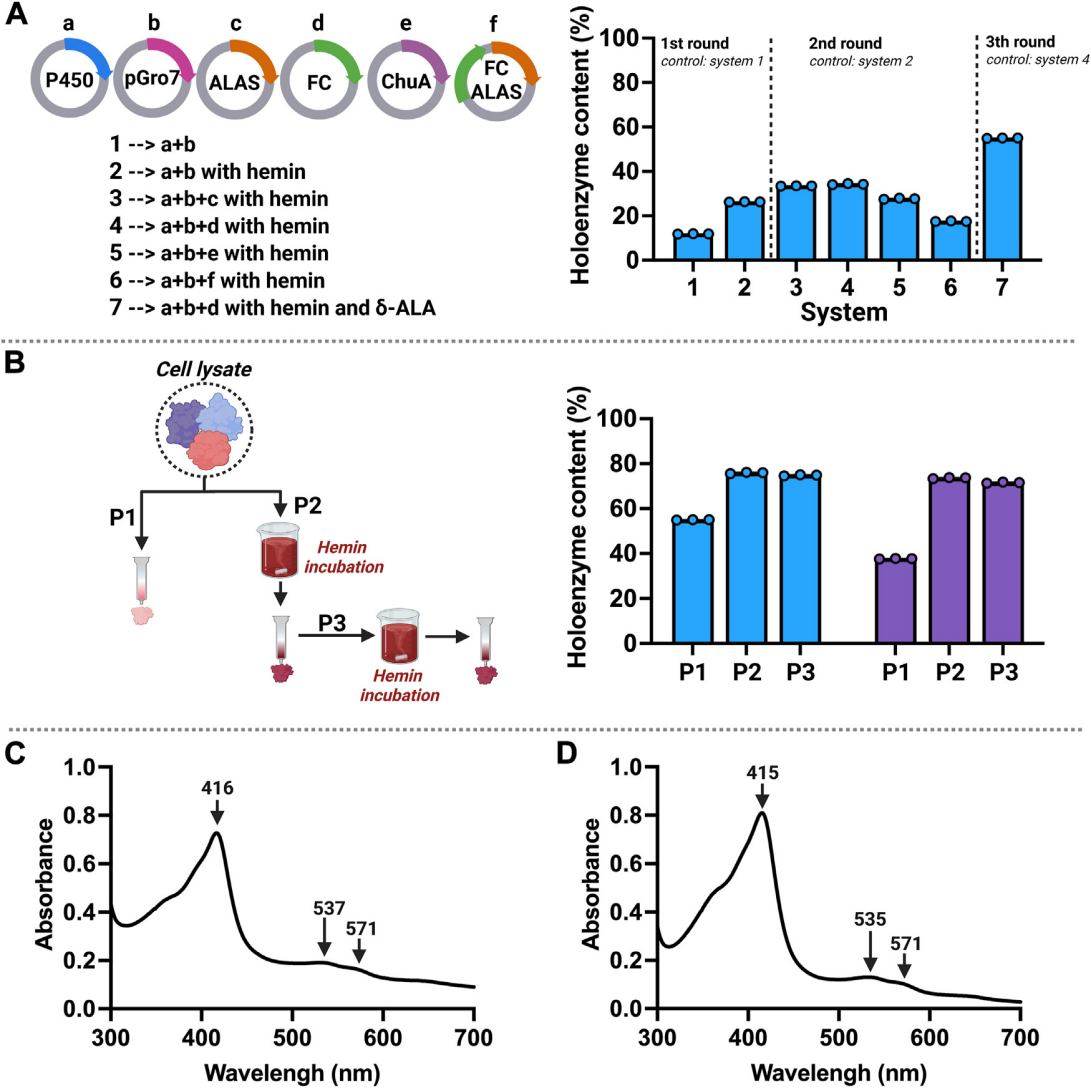
**Improvement of overexpression and purification of CYP152 holoenzymes.***A*, experimental design to improve production of holoenzyme of OleT_LA_ using Systems 1 to 7 which co-expressed additional genes encoding for proteins related to heme synthesis. The plasmids used included: a, CYP152 gene in the pET-22b(+) vector; b, *Gro*EL and *Gro*ES in the pGro7 vector; c, d, e, f are genes encoding ALA synthase (ALAS), ferrochelatase (FC), outer membrane heme recepter (ChuA), and FC and ALAS, respectively, in the pRSFDuet−1 vector. Details of the results are described in the Supplementary results (Fig. S3). *B*, the cell lysates of OleT_LA_ and OleT_JE_ which was cultured using the overexpression System 7, were used for protein purification using a nickel nitriloacetic acid column without extra hemin addition (Method P1), or with hemin addition and incubation for 16 h before purification (Method P2). The purified enzyme from P2 was further incubated with additional hemin for 16 h before removing free hemin (Method P3). The holoenzyme content (%) was calculated based on the percentage of the enzyme-bound heme per the total amount of purified enzyme. *C*, an absorption spectrum of the purified OleT_LA_ overexpressed using System 7 and addition of hemin (Method P2). *D*, an absorption spectrum of the purified OleT_JE_ overexpressed using System 7 and addition of hemin (Method P2). SD values were reported from three replications of each analysis.

In this study, we comprehensively tested the effects of hemin, co-expression systems, and δ-ALA using three rounds of experiments (7 systems) on the production of CYP152 (OleT_LA_) holoenzymes using ZY auto-induction medium for cell growth (Details in Fig. S3). The results indicated that *System 7* in which the OleT_LA_ plasmid was co-expressed with ferrochelatase and pGro7, and in which the culture medium was supplemented with δ-ALA and hemin, provided the highest holoenzyme yield (54.9% of the total purified holoenzyme) among all systems tested (Fig. 3*A*). We also tested the effects of δ-ALA supplementation alone, ferrochelatase expression alone, and the combination of δ-ALA supplementation and ferrochelatase expression without hemin. The resulting holoenzyme contents were 30.4 ± 0.9%, 29.7 ± 1.5%, and 48.8 ± 0.8%, respectively, which were lower than that observed in System 7. These results indicate that the combination of δ-ALA and hemin supplementation, along with co-expression of ferrochelatase, is necessary for improving CYP152 holoenzyme expression.

To further increase the heme content, we used *System 7* for holoenzyme production and tested whether the addition of hemin into crude protein lysate before purification could increase the amount of holoenzyme for both OleT_LA_ and OleT_JE_. The results showed that the purification without adding extra hemin (*Method P1*) of OleT_LA_ and OleT_JE_ yielded 55.0% (119.9 mg of purified holoenzyme/L of culture medium), and 37.7% (107.4 mg of purified holoenzyme/L of culture medium), respectively. The addition of hemin (*Method P2*) yielded 75.9% (178.1 mg of purified holoenzyme/L of culture medium) and 73.1% (171.5 mg of purified holoenzyme/L of culture medium) of holoenzyme production for OleT_LA_ and OleT_JE_, respectively (Fig. 3*B*, Table S1). From these results, it is clear that the *P2* purification method is superior to *P1* because it could improve the holoenzyme content for both OleT_LA_ and OleT_JE_ by 1.4-fold and 1.9-fold, respectively. These results indicate that the overexpressed OleT_LA_ and OleT_JE_ in apoenzyme form can bind with exogenous hemin to result in the formation of holoenzyme in the crude lysate. To ensure that hemin was fully bound to the protein, we performed another incubation with hemin after purification by the *P2* method (*Method P3*). The results showed that the levels of bound heme were not increased as compared to the *P2* method (Fig. 3*B*, Table S1), indicating that a single-step (*Method P2*) of reconstitution is sufficient for complete heme reconstitution. Altogether, our results indicate that the best system for holoenzyme production of CYP152 enzymes *i.e.,* OleT_LA_ and OleT_JE_ is *System 7* for overexpression with the addition of hemin in the crude protein lysate before purification.

To the best of our knowledge, the conditions identified for producing CYP152 holoenzymes described earlier is the best system reported to date for the enzymes in the CYP152 family. When compared to previous reports of OleT_JE_ holoenzyme production, our conditions gave higher holoenzyme production yields of around 8.6-fold and 114.3-fold compared to those of OleT_JE_ wild-type and variant (R245E), respectively (36). Our system also showed higher production than that of the P450_Jα_ system, which was the system with the highest holoenzyme production yield of around 3.5-fold (16).

Sodium dodecyl sulfate-polyacrylamide gel electrophoresis (SDS-PAGE) analysis of the purified OleT_LA_ and OleT_JE_ showed protein bands with molecular weights of 49 kDa which corresponded to both OleT_LA_ and OleT_JE_ (Fig. S4). Absorption spectra of the purified OleT_LA_ and OleT_JE_ also showed characteristic peaks of CYP152 enzymes at 416, 537, and 571 nm (Fig. 3*C*), and at 415, 535, and 571 nm (Fig. 3*D*), respectively. The characteristic spectra demonstrates that both enzymes bind heme cofactors properly. The amount of enzyme used in further experiments was quantitated based on a holoenzyme concentration measured using pyridine hemochromagen assay (40).

### Investigation of enzyme stability

**Figure 4:**
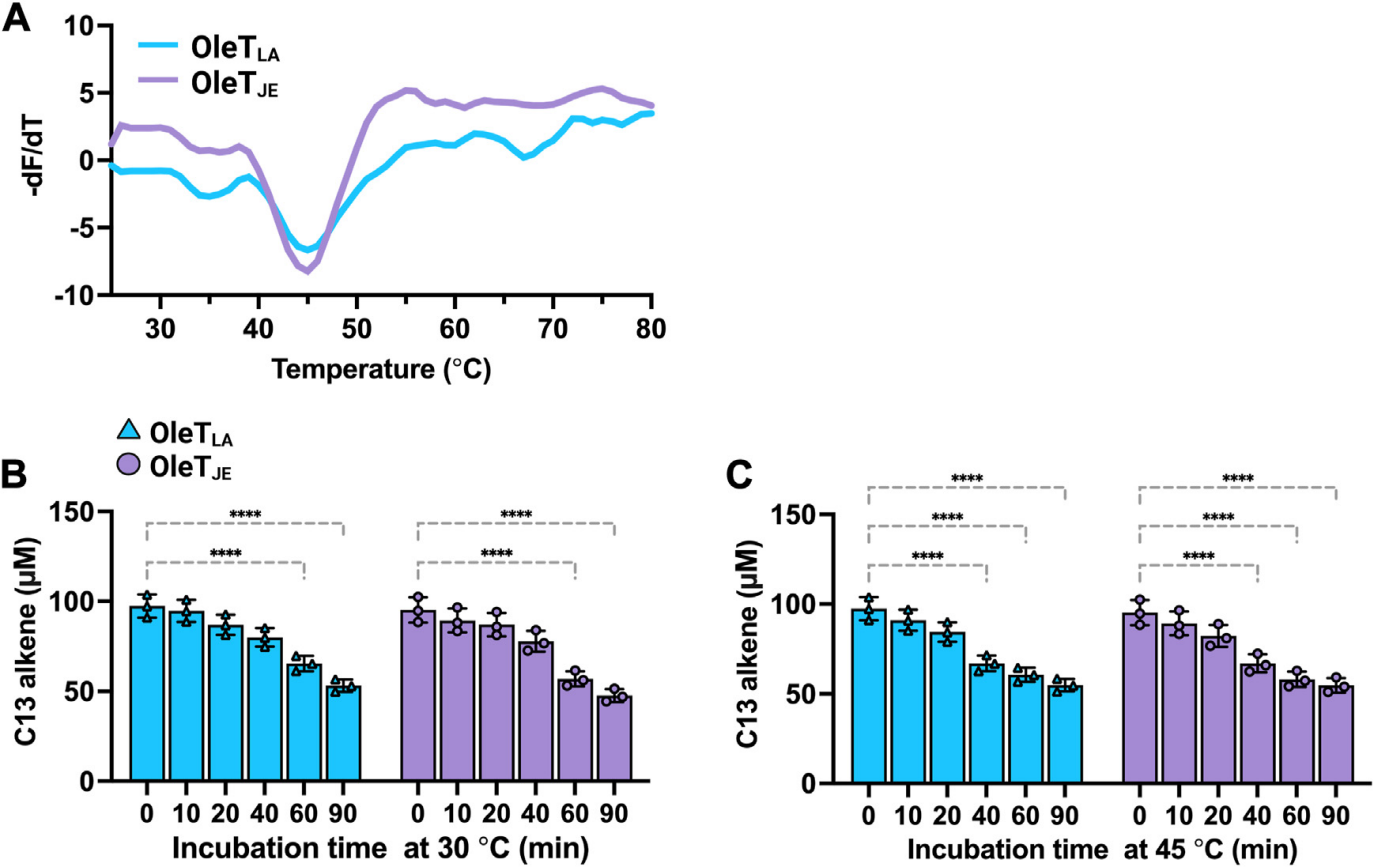
**Thermostability of OleT_LA_ and OleT_JE_**. *A*, measurement of melting temperatures of OleT_LA_ and OleT_JE_ using thermofluor assays. *B*, thermostability of OleT_LA_ and OleT_JE_ at 30 °C. *C*, thermostability of OleT_LA_ and OleT_JE_ at 45 °C. Results are shown as the mean ± SD (n = 3). *Asterisks* denote statistical significances (∗∗∗∗*p* ≤ 0.001) as determined by two-way ANOVA followed by multiple comparison test.

Thermofluor assays (details described in Experimental Procedures) were used to measure the melting temperatures of OleT_LA_ and OleT_JE_ in 50 mM NaH_2_PO_4_, 300 mM NaCl, pH 7.5. Both OleT_JE_ and OleT_LA_ showed melting temperatures around 45 °C (Fig. 4*A*). Additionally, the thermostability of both enzymes at 30 °C and 45 °C (Fig. 4, *B*, *C*) were investigated. The results indicate that both enzymes exhibited very similar thermostabilities at both temperatures. At 30 °C, both of them can maintain 100% activities for up to 40 min, and at 45 °C for up to 20 min. After the time period mentioned, the decarboxylation activities of both enzymes significantly dropped. We noted small discrepancies in the melting temperature of OleT_JE_ measured in this study which was slightly lower than the previously reported value of 48.6 °C (in 100 mM potassium phosphate, 750 mM NaCl, 10% glycerol at pH 8.0) (10). This may be due to the difference in buffers used.

### Optimization of bioconversion conditions

**Figure 5:**
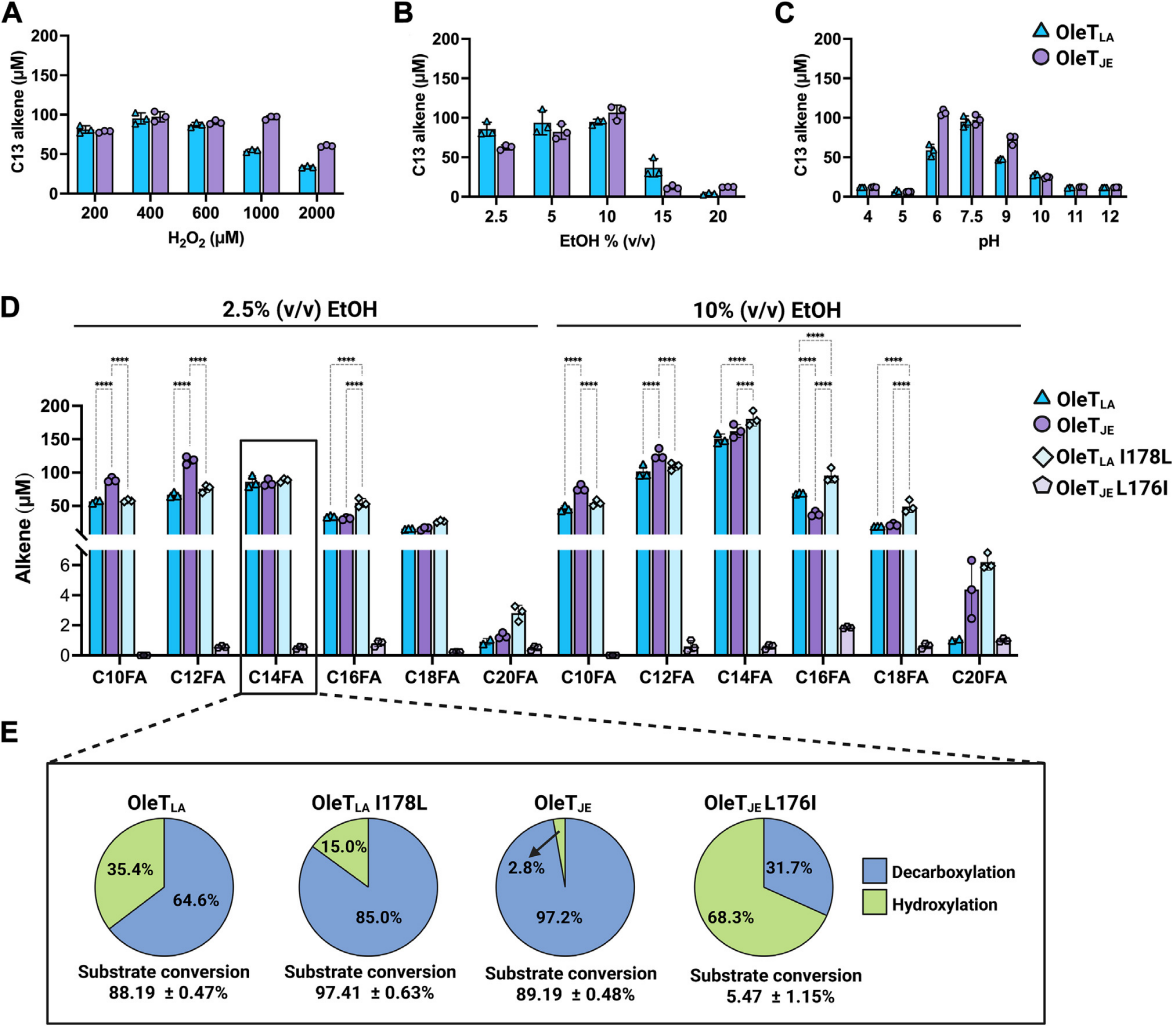
**Optimization of decarboxylation reactions and decarboxylation activity.** The enzymatic reactions of OleT_LA_ and OleT_JE_ were carried out under various *A*, H_2_O_2_ concentrations, *B*, EtOH concentrations, and *C*, pH values. *D*, alkene production yields from the OleT_LA_, OleT_JE_, OleT_LA_ I178L, and OleT_JE_ L176I reactions using fatty acids with various chain lengths from C10FA to C20FA in 2.5% (v/v) EtOH (*left panel*) and in 10% (v/v) EtOH (*right panel*) as co-solvent. All reactions contained 5 μM enzyme, 200 μM of substrate and 400 μM of H_2_O_2_. The reactions were performed at 25 °C for 1 h and then quenched by adding two-fold volume of ethyl acetate. Alkene product analysis was done by GC-MS. Results are shown as the mean ± SD (n = 3). *Asterisks* denote statistical significances (∗∗∗∗*p* ≤ 0.001) as determined by two-way ANOVA followed by multiple comparison tests. *E,* Percentage of decarboxylation and hydroxylation products, and substrate conversion by OleT_LA_, OleT_JE_, and their variants. Reactions contained 5 μM of each enzyme, 200 μM of C14FA, 400 μM of H_2_O_2_ in 50 mM of NaH_2_PO_4_, 300 mM NaCl pH 7.5. The reactions were performed at 25 °C for 1 h. Results are shown as the mean ± SD (n = 3). The product percentage yield was calculated based on the amount of substrate utilized. Decarboxylation was calculated based on the amount of alkene formed while hydroxylation was determined from the total amount of α-hydroxy and β-hydroxy fatty acids formed.

To optimize the enzymatic reactions for each enzyme, we first used C14FA at a fixed concentration of 0.2 mM and varied the concentration of H_2_O_2_ (Fig. 5*A*). OleT_JE_ showed the highest alkene production yield when H_2_O_2_ was used in a range of 400 to 1000 μM, while OleT_LA_ yields the highest alkene production in a range of 400 to 600 μM of H_2_O_2_. When using 400 μM of H_2_O_2_, both OleT_LA_ and OleT_JE_ showed similar bioconversion yields. Therefore, in the subsequent experiments for both enzymes, we used 400 μM of H_2_O_2_ and 200 μM of fatty acid substrates to investigate the effects of solvent and pH on bioconversion as high H_2_O_2_ concentrations are not practical for real bioconversion applications. Due to difficulties in dissolving a substrate in aqueous solutions, EtOH was added into the system at a concentration range of 2.5 to 20% (v/v). The decarboxylation product was found to be maximal when using 10% (v/v) EtOH for both enzymes (Fig. 5*B*). For pH preference, the optimal pH for both enzymes was 7.5 (Fig. 5*C*).

### Analysis of decarboxylation activities with fatty acids of various chain lengths using H_2_O_2_ as a co-substrate

Based on the conditions obtained from the previous experiments, we further explored bioconversion and the products generated by the reactions of OleT_LA_ and OleT_JE_ and fatty acids with different carbon chain lengths from 10 carbon to 20 carbon atoms. The reactions contained 200 μM of fatty acid and 400 μM of H_2_O_2_ at pH 7.5. For EtOH, because OleT_LA_ and OleT_JE_ can tolerate up to 10% (v/v) of EtOH, we thus carried out the experiments using 2.5% (v/v) and 10% (v/v) of EtOH as a co-solvent. We expected that 10% (v/v) of EtOH might give a better yield because it could increase substrate solubility.

The results clearly showed that OleT_JE_ could use decanoic acid (C10FA) and C12FA as substrates to produce alkenes with higher yield than those of OleT_LA_ under both 2.5 and 10% (v/v) of EtOH. For C14FA, both enzymes exhibited no significant difference in alkene production yield at 2.5% (v/v) EtOH. However, OleT_JE_ showed slightly higher C13 alkene formation than OleT_LA_ at 10% (v/v) EtOH (Fig. 5*D,* S5). However, for the C16FA reaction under 10% (v/v) of EtOH, OleT_LA_ generates more alkene than the reaction of OleT_JE_ by 1.8-fold. These results indicate that OleT_JE_ prefers to use shorter-chain fatty acids, whereas OleT_LA_ can use longer chain fatty acids such as C16FA better than OleT_JE_. The results for OleT_JE_ are also consistent with those of the previous studies (12, 19, 20). Interestingly, 10% (v/v) EtOH could significantly enhance the overall alkene production yields in the reactions of OleT_LA_ with C12FA, C14FA, C16FA, and C18FA, while it could improve decarboxylation of OleT_JE_ only in the reaction with C14FA (Fig. S6).

The product distribution in OleT_LA_ and OleT_JE_ reactions were analyzed using C14FA and C16FA as substrates. The results showed that both OleT_JE_ and OleT_LA_ catalyze reactions to generate alkenes as major products for both substrates. However, OleT_LA_ generates more β-hydroxy C14FA and α-hydroxy C14FA than OleT_JE_ when using C14FA as a substrate (Fig. S7*A*). No differences in product distribution were observed in either enzyme when using C16FA as substrate, with both enzymes generating only small amounts of β-hydroxy C16FA (Fig. S7*B*). Our results for the OleT_JE_ reactions were consistent with prior studies which showed that OleT_JE_ generates terminal alkene as a major product and β-hydroxy fatty acid as a minor product (12, 19, 20, 25). For the reactions with C10FA and C12FA, OleT_LA_ generated more hydroxylated products than OleT_JE_ (analyzed by chromatogram). HPLC-MS chromatograms of selected ion monitoring mode (SIM mode) of hydroxylated products showed five peaks in the OleT_LA_ reaction when using C10FA (Fig. S8*A*) and C12FA (Fig. S8*B*) as substrates. While OleT_JE_ displayed only two peaks of hydroxylated products of C10FA (Fig. S8*A*) and one peak of hydroxylated products of C12FA (Fig. S9). For the reaction with C18FA, OleT_LA_ showed one peak of hydroxylated product, while OleT_JE_ did not show any peaks (Fig. S8*C*). These results also indicate that OleT_LA_ produces a variety of hydroxy fatty acids, not only β-hydroxy and α-hydroxy fatty acids, but possibly γ, δ, and ε-hydroxy fatty acids, similar to the reactions catalyzed by CYP152_MP_ (17) and the L176G variant of OleT_JE_ (30). For icosanoic acid (C20FA), both enzymes did not show any peaks corresponding to hydroxylated products in their chromatograms.

### Steady-state kinetics

C16FA was thus further selected as a substrate for investigating and comparing the steady-state kinetics of OleT_LA_ and OleT_JE_ in 10% (v/v) of EtOH. Results of steady-state kinetic analysis under these conditions are shown in Table 1 and Figure S9. The *k*_cat_ value of C16FA in 10% (v/v) of EtOH was significantly greater than that of the same reaction in the presence of 2.5% (v/v) of EtOH for both OleT_LA_ and OleT_JE_. Overall, the presence of 10% (v/v) of EtOH showed an enhancement of around 4.3-fold for OleT_LA_ and 1.4-fold for OleT_JE_ when compared to the reaction in the presence of 2.5% (v/v) of EtOH. The kinetic parameters clearly indicate that using 10% (v/v) EtOH can improve the catalytic activity of OleT_LA_ significantly.

**Table 1:** Kinetic parameters of C16FA decarboxylation catalyzed by OleT_LA_, OleT_JE_ and the OleT_LA_ I178L variant

Enzyme	EtOH % (v/v)	*k*_cat_ (min^−1^)	*K*_M_ (μM)	*k*_cat_/*K*_M_ (min^−1^ μM^−1^)
OleT_LA_	2.5	20.6 ± 0.5	5.4 ± 0.6	3.8 ± 0.5
OleT_LA_	10	88.4 ± 1.6	16.7 ± 0.6	5.3 ± 2.1
OleT_JE_	2.5	38.8 ± 0.9	8.6 ± 1.4	4.6 ± 0.6
OleT_JE_	10	55.1 ± 0.2	9.6 ± 0.6	5.7 ± 0.4
OleT_LA_ I178L	10	96.0 ± 2.5	25.0 ± 0.6	3.8 ± 0.2

Details of experiments were described in Experimental Procedures. Results are shown as the mean ± SD (n = 3).

### Measurement of substrate binding affinity by isothermal titration calorimetry (ITC)

To evaluate the thermodynamics of substrate binding, we investigated the binding of C16FA to OleT_LA_ compared to that of OleT_JE_ (Experimental Procedures). The measurements were carried out in two systems including 2.5% (v/v) and 10% (v/v) of EtOH. The results are shown in Table 2 and Figure S10. The *K*_d_ for binding of C16FA to OleT_LA_ was measured as 0.95 and 1.26 μM under the conditions in the presence of 2.5% and 10% (v/v) of EtOH, respectively. The data indicate a high-affinity binding between OleT_LA_ and C16FA, and the presence of 10% (v/v) of EtOH does not influence much of the enzyme and substrate interactions. Moreover, positive enthalpy changes of 29.97 kcal/mol and 50.83 kcal/mol were detected for the conditions under 2.5% and 10% (v/v) of EtOH, respectively. Positive enthalpy changes indicate that the binding is entropy-driven, implying that the binding between enzyme and substrate is dominated by hydrophobic interactions, enzyme conformation changes (42), or rearrangement of enzyme-bound water molecules (43). The increased enthalpy changes in the presence of 10% (v/v) of EtOH indicate greater conformation change and/or greater hydrophobic stabilization during the substrate binding process under this condition. The root cause of conformational changes of OleT_LA_ in the presence of EtOH was later investigated by molecular dynamic simulations (see results later).

**Table 2:** Substrate binding parameters of C16FA to OleT_LA_ and OleT_JE_

Enzyme	EtOH % (v/v)	*K*_d_ (μM)	ΔH (kcal/mol)	ΔG (kcal/mol)	TΔS (kcal/mol)
OleT_LA_	2.5	0.95 ± 0.14	29.97 ± 2.93	−8.22 ± 0.08	38.10 ± 2.65
OleT_LA_	10	1.26 ± 0.23	50.83 ± 2.10	−8.06 ± 0.11	58.93 ± 2.00
OleT_JE_	2.5	1.93 ± 0.16	8.33 ± 2.14	−7.78 ± 0.05	16.13 ± 2.15
OleT_JE_	10	0.44 ± 0.01	−12.19 ± 5.11	−8.67 ± 0.01	−5.15 ± 2.74

All experiments were performed at 25 °C. Both C16FA and enzyme were dissolved in 50 mM NaH_2_PO_4_ buffer pH 7.5 with 300 mM NaCl and 2.5% (v/v) EtOH or 10% (v/v) EtOH. In the binding measurements, we used 100 μM of OleT_LA_, and 200 μM OleT_JE_ because the signals were low when using 100 μM OleT_JE_. Results are shown as the mean ± SD (n = 3).

For OleT_JE_, *K*_d_ values for binding of C16FA in the presence of 2.5% (v/v) and 10% (v/v) EtOH were measured as 1.93 μM and 0.44 μM, respectively. The data indicate that under the 2.5% (v/v) of EtOH condition, C16FA binds tighter to OleT_LA_ than OleT_JE_, while in the presence of 10% (v/v) EtOH, C16FA binds tighter to OleT_JE_. This greater difference in *K*_d_ values under different EtOH concentrations also implies that the presence of 10% (v/v) EtOH affects the enzyme and substrate interactions much more than in the case of OleT_LA_. Interestingly, the presence of 10% of EtOH also affects the enthalpy changes (ΔH) of C16FA binding, going from positive to negative values when the EtOH concentration is increased 2.5% (v/v) to 10% (v/v). The data clearly imply that upon increasing EtOH, the C16FA and OleT_JE_ binding nature changed from hydrophobic interactions and water rearrangement to be enthalpy-driven binding.

### The X-ray structure of OleT_LA_ with C20FA bound

To understand the structural feature governing the biophysical and biochemical properties of OleT_LA_ that are different from those of OleT_JE_, we co-crystallized OleT_LA_ with C20FA (PDB: 9JQM) for comparing with the OleT_JE_ structure co-crystallized with C20FA (PDB: 4L40) previously reported. Details of data collection and refinement are presented in Table 3. Although the overall architecture of OleT_LA_: C20FA is similar to that of OleT_JE_: C20FA, we noted distinct differences between the two enzymes. Comparion of Chain A and Chain B of OleT_LA_ with those of OleT_JE_ yielded root mean square deviations (RMSD) of 0.413 (345–345 atoms) and 0.384 (340–340 atoms), respectively.

**Table 3:** Crystallographic data and refinement statistics

Parameters	OleT_LA_: Icosanoic acid (C20FA)
Data Collection	
X-ray source	SPring-8 BL44XU
Wavelength (Å)	0.9000
Space group	*I*2_1_2_1_2_1_
Unit-cell parameters	
(Å)	*a* = 82.80, *b* = 188.86, *c* = 198.31
(°)	β = 90.00
Resolution (Å)	42.63–2.44 (2.59–2.44)
Number of unique reflections	57,908 (9086)
*R*_merge_	0.076 (0.783)
Completeness (%)	99.6 (97.9)
Mean *I*/σ (*I*)	13.51 (1.62)
CC1/2	0.998 (0.791)
Redundancy	6.68 (6.89)
Refinement	
*R*_work_/*R*_free_	0.182/0.230
Number of atoms	
Protein	6906
Ligands	130
Water	557
B-factors (Å^2^)	
Protein	70.25
Ligands	62.63
Water	75.44
Average B-factor	70.54
Ramachandran plot statistics	
Favored (%)	96.1
Allowed (%)	3.7
Outliers (%)	0.2
R.m.s. deviations	
Bonds (Å)	0.009
Angles (°)	1.017
PDB code	9JQM

Values for the highest resolution shell are shown in parentheses.

**Figure 6:**
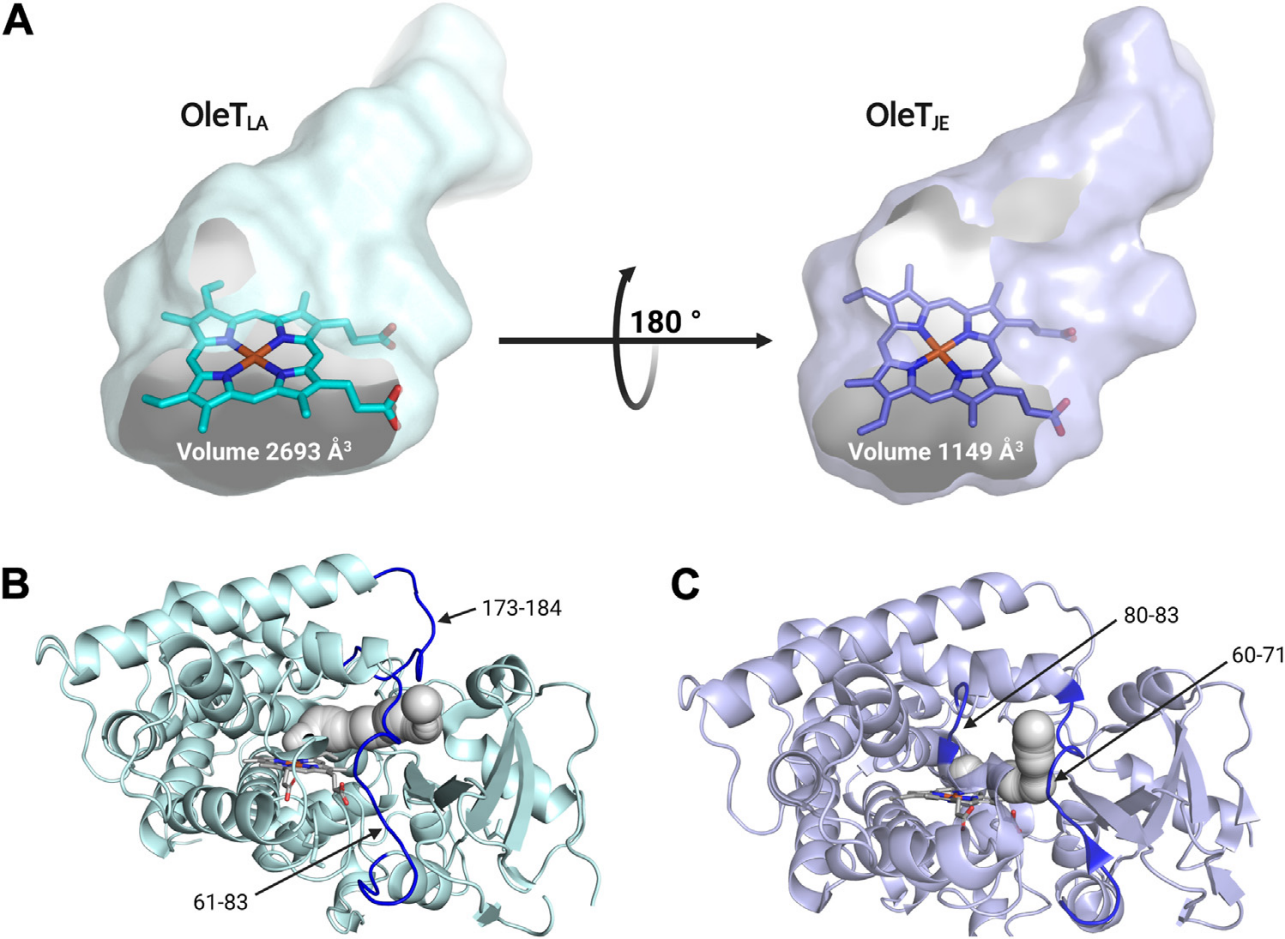
**Difference in the heme configuration and active site binding pockets of OleT_LA_ and OleT_JE_**. *A,* the difference in heme binding configuration between OleT_LA_ (*light blue stick*), and OleT_JE_ (*purple stick*). Illustration of substrate tunnels and measurement of the cavity size of OleT_LA_ and OleT_JE_ as 2693 Å^3^ and 1149 Å^3^, respectively. *B,* the main substrate tunnel (*gray sphere*) of OleT_LA_ points towards between the two loops of residues 173 to 184 and 61 to 83. *C,* the main substrate tunnel (*gray sphere*) of OleT_JE_ points towards the two loops of residues 60 to 71 and 80 to 83.

Significant distinctions were observed in the FG-loop region (residue 173–175 of OleT_LA_) and the β-sheet (residue 388–391 and 413–419 of OleT_LA_) at the C-terminal (Fig. S11, *A* and *B*). The electron density in these regions, particularly at the FG-loop region, was less defined, indicating greater flexibility in the OleT_LA_ structure compared to OleT_JE_. OleT_LA_ is notably different from OleT_JE_ and almost all other CYP152s in its heme orientation. OleT_LA_ displays a “flipped” heme orientation (Fig. 6*A*) which was only observed in CYP152N1 (PDB: 5YHJ) (15). However, catalytic properties related to the flipped heme of CYP152N1 were not well documented. For OleT_LA_, although flipped heme was observed, there was no significant difference in protein expression compared to the usual heme-binding enzyme (OleT_JE_).

We also analyzed residues surrounding the heme-binding region and the active site cavities to identify the cause of this flipped heme configuration (Fig. S12). The analysis suggests that the flipped-heme configuration may occur in enzymes with large active site cavities. The cavity sizes of OleT_LA_ and CYP152N1, which have unusual heme binding, were measured as 2693 Å^3^ and 2637 Å^3^, respectively, whereas OleT_JE_ which has normal heme binding showed cavities of 1149 Å^3^. Similarly, other enzymes with normal heme binding, such as CYP152K6, CYP152A1, and CYP152B1, displayed cavity sizes ranging from 1548 Å^3^ to 2311 Å^3^ (Table S2). The unusual heme binding has also been reported in cytochrome *b*_5_, where different forms exhibited different redox potentials. However, this does not affect its function (44). Additionally, the flipped heme binding has been observed in myoglobin. A different form of heme binding may be a cause for the difference in unfolding and refolding rates (45). In other cytochrome P450-dependent enzymes, the unusual heme binding has been observed in CYP105P2 (PDB: 5IT1) (46), CYP121A1 (PDB: 1N40) (47), CYP154A1 (PDB: 1ODO) (48), and the OxyA homolog from Kistamicin biosynthesis (OxyAkis, PDB: 7TTO) (49). For OxyAkis, a flipped heme was found in a specific batch of enzyme preparation, while other batches displayed the usual binding mode.

To gain insights into the difference in substrate recognition between the two enzymes, we further analyzed the substrate tunnel of OleT_LA_ and compared it with OleT_JE_. The OleT_LA_ structure with C20FA bound (PDB: 9JQM) and the OleT_JE_ structure with C20FA bound (PDB: 4L40) were used for analysis of tunnels connecting from the outside to the heme-binding site. Three tunnels of OleT_LA_ including the main substrate tunnel pointing towards the FG-loop (residues 173–184) and the loop with residues 61 to 83 (Fig. 6*B*) while four tunnels of OleT_JE_ including the main substrate tunnel pointing toward other two loops (residues 60–71, and 80–83) were identified (Fig. 6*C*). Notably, the FG-loop-directed main substrate tunnel of OleT_LA_ differs from the tunnel directions observed in other CYP152s with predominant decarboxylation activity including OleT_JE_. We hypothesize that the substrate tunnel orientation toward the FG-loop in OleT_LA_ may be influenced by high FG-loop flexibility (see MD results later). The configuration of a substrate tunnel toward the FG-loop is similar to those observed in CYP152 fatty acid hydroxylases such as CYP152A1 (PDB: 1IZO) (50), CYP152B1 (PDB: 3AWM) (13), CYP152K6 (PDB: 6FYJ) (14), and CYP152N1 (PDB: 5YHJ) (15); however, OleT_LA_ still exhibits predominant of decarboxylation. These structural and activity profiles of OleT_LA_ imply that the configuration of the substrate tunnels of CYP152 enzymes is not a major factor controlling decarboxylation or hydroxylation activities.

As salt bridge networks, hydrophobic clusters, charged residues, and hydrophobic residues are known to affect biophysical properties of P450 enzymes (51, 52), we thus further analyzed these mentioned parameters of OleT_LA_ and compared them to those of OleT_JE_. For charged residues, OleT_LA_ and OleT_JE_ showed 30.66% mol and 29.15% mol content, respectively; this may attribute to the similar thermostability and melting temperatures observed between the two enzymes. When the protein structures were examined for their salt bridge networks and hydrophobic clusters using ProteinTools (53), the results revealed the same number of salt bridges for both OleT_LA_ and OleT_JE_. However, the two enzymes are notably different in the number of hydrophobic clusters in which 13 clusters were found for OleT_LA_ and 9 clusters for OleT_JE_. These data imply that the hydrophobic interactions within the structure of OleT_LA_ are greater than those of OleT_JE_. The high degree of hydrophobic clusters was proposed to be attributable to solvent tolerance properties of ancestral CYP3A4 (CYP3_N1) that retained 91% of activity in 10% (v/v) of methanol (54). Therefore, a higher number of hydrophobic clusters in OleT_LA_ may contribute to its higher tolerance to 10% (v/v) EtOH and thus greater decarboxylation activity of C16FA than OleT_JE_ (Fig. 5*D*, Table 1).

### Identification of OleT_LA_ structural features promoting C16FA decarboxylation and solvent tolerance

**Figure 7:**
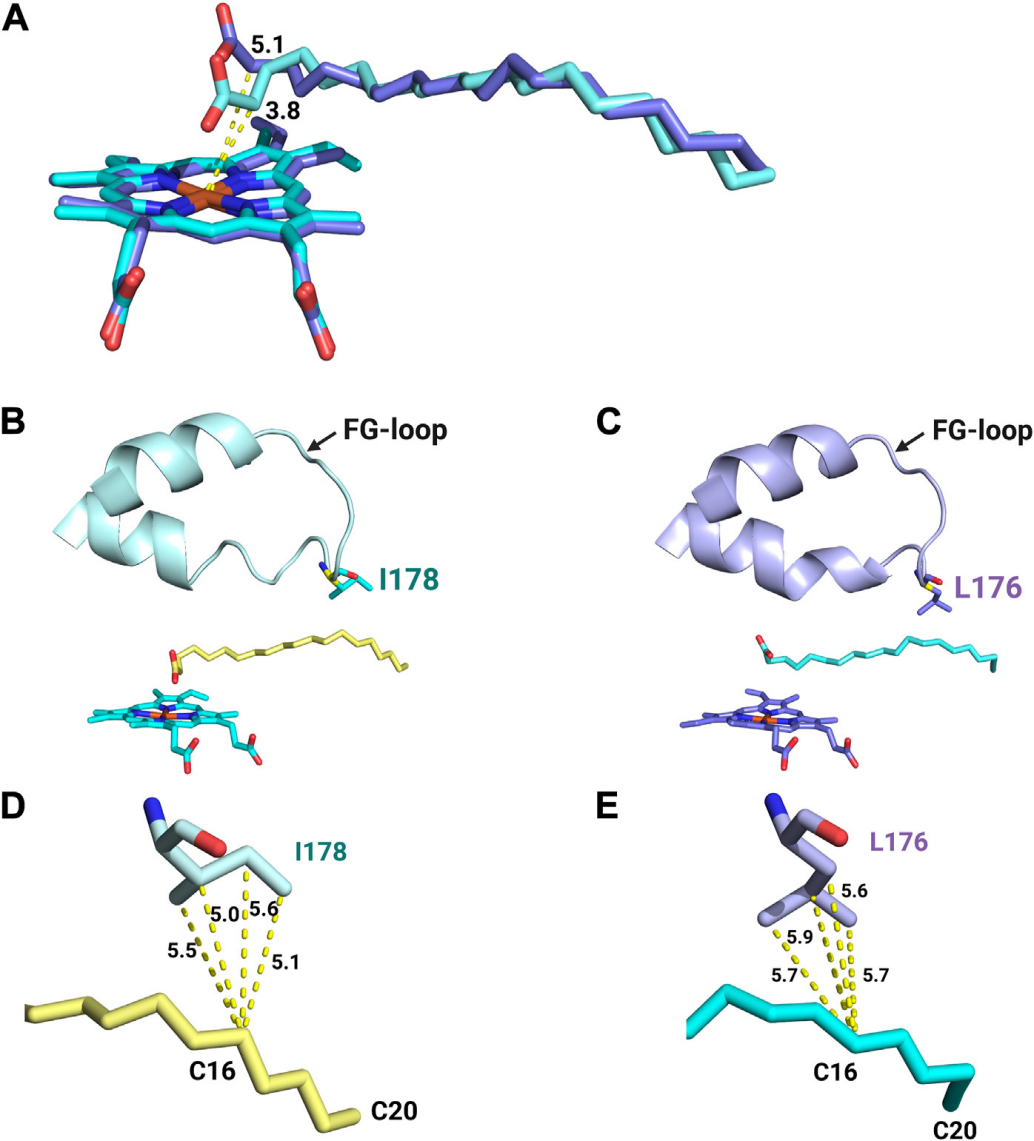
**Comparison of substrate binding and active site residues of OleT_LA_ and OleT_JE_**. *A*, overlaid of icosanoic acid bound in the active site of OleT_LA_ (*blue stick*) and OleT_JE_ (*purple stick*). The distance between alpha carbon and heme iron center in the two enzymes are significantly different. *B*, configuration of the FG-loop of OleT_LA_ and I178 located at the FG-loop region. *C,* configuration of the FG-loop of OleT_JE_ and L176 located at the FG-loop region. *D*, distance between the side chain carbons of I178 and C16 atom of C20FA in OleT_LA_. *E*, distance between the side chain carbons of L176 and C16 atom of C20FA in OleT_JE_.

We further analyzed Chain B of OleT_LA_ which binds C20FA in comparison to the C20FA binding in OleT_JE_ (Fig. S13*A*). In both structures, almost all residues surrounding C20FA are similar in the two enzymes except V76 (I74 in OleT_JE_), Y296 (F294 in OleT_JE_), I178 (L176 in OleT_JE_), I50 (V48 in OleT_JE_), and I16 (V16 in OleT_JE_). Other conserved residues including H87 (H85 in OleT_JE_), R247 (R245 in OleT_JE_), and C367 (C365 in OleT_JE_) exhibit good alignment between the two enzymes (Fig. S13, *B, C*). Another key difference between the two enzymes is on the substrate binding configuration, particularly at the carboxylic and C_α_ regions of the substrate (Fig. 7*A*). This discrepancy may be attributed to the flexibility observed in the helix and FG-loop (residues 153–178) lining the substrate binding site in OleT_LA_ (Fig. 7, *B* and *C*). Notably, I178 in the FG-loop of OleT_LA_ (equivalent to L176 in OleT_JE_) exhibits a shorter distance to C16 atom of C20FA than does L176 of OleT_JE_ (Fig. 7, *D* and *E*). We thus speculated that I178 may enhance the interactions around the substrate C16 position and thus promote greater decarboxylation activity in OleT_LA_ compared to OleT_JE_ when C16FA was used as a substrate (Table 1). This residue was thus investigated for its roles in the two enzymes by site-directed mutagenesis (see Results later).

### Molecular dynamics simulations to explain EtOH tolerance of OleT_LA_

As the reaction of OleT_LA_ with C16FA is much faster in the presence of 10% (v/v) of EtOH, and OleT_LA_ is much more stable than OleT_JE_ under this condition, we thus used molecular dynamics (MD) simulations to investigate the effects of EtOH on the overall structural rigidity/flexibility of both enzymes.

**Figure 8:**
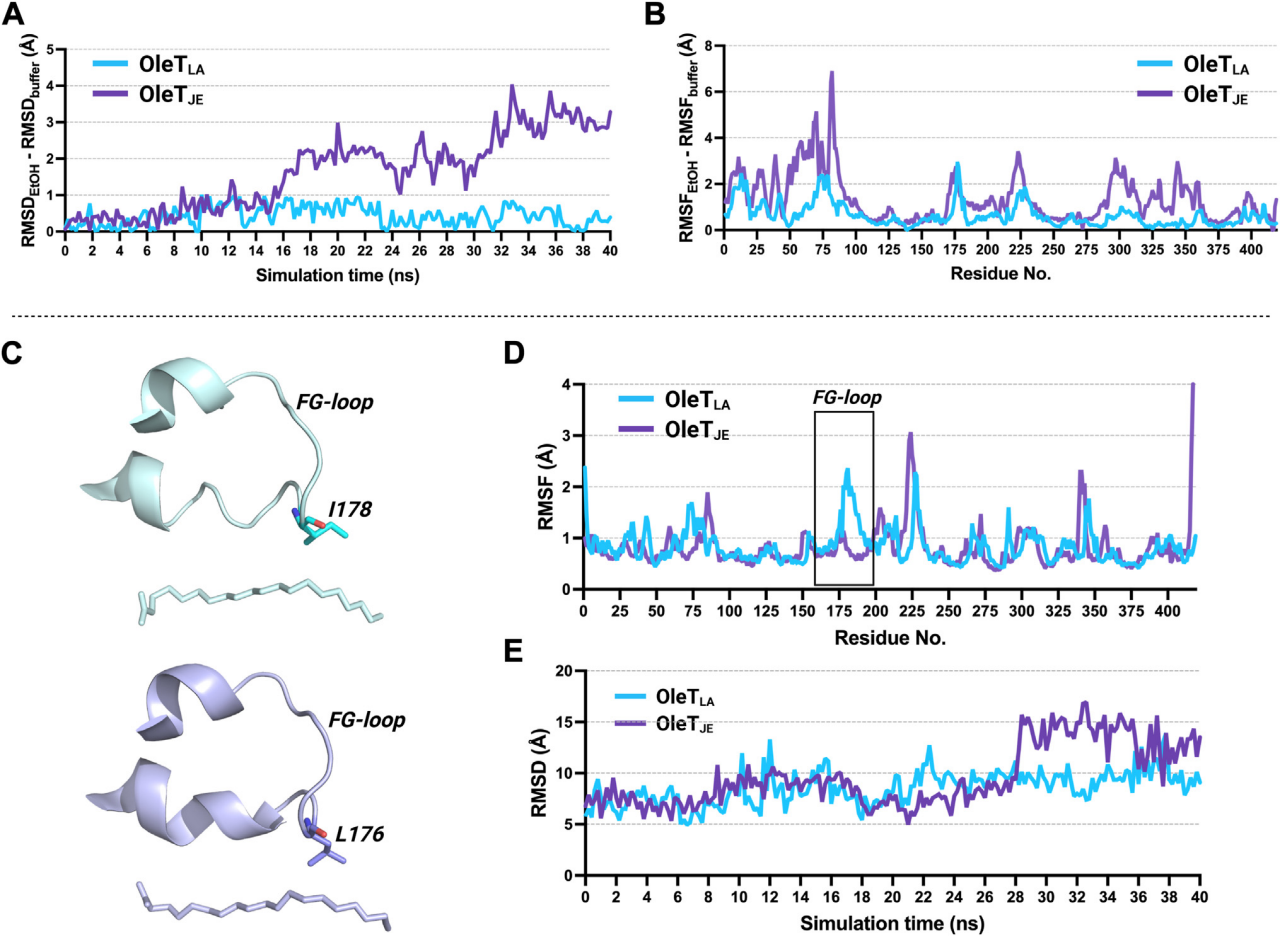
**Molecular dynamics simulations of OleT_LA_ (PDB:**9JQM**) and OleT_JE_ (PDB:**4L40**).***A,* the difference between RMSD in EtOH and buffer systems of OleT_LA_ and OleT_JE_. *B,* the difference between RMSF in EtOH and buffer systems of OleT_LA_ and OleT_JE_. The simulations were performed at 380 K for 40 ns of the total simulation time. *C,* the structure of FG-loop, I178 and C20FA in the active site of OleT_LA_ (*above*), and the structure of FG-loop, L176 and C20FA in the active site of OleT_JE_ (*below*). *D,* the RMSF values of OleT_LA_ (*blue line*), and OleT_JE_ (*purple line*) at 380 K were obtained from 40 ns MD simulations in buffer system. *E,* distances between C16 atom of C20FA *versus* the C_α_ of I178 in OleT_LA_ (*blue line*) or L176 of OleT_JE_ (*purple line*) calculated from RMSD values obtained from 40 ns MD simulations in buffer system.

The root mean square deviation (RMSD) values calculated for the C_α_ backbone of all residues were used to investigate the overall rigidity of the entire structures of both OleT_LA_ and OleT_JE_ in buffer and EtOH systems. The results indicate that during MD simulations (40 ns), the different RMSD values of OleT_LA_ in buffer and EtOH systems are less than 1 Å, implying that the OleT_LA_ structure remains stable in the presence of EtOH. On the contrary, the different RMSD values of OleT_JE_ deviate around 3.5 Å over 40 ns of the simulation time (Fig. 8*A*). In addition, the regions contributing to the structural stability of each enzyme were identified using root mean square fluctuation (RMSF) to examine fluctuation regions in both enzymes.

The results showed that when comparing the conditions with and without EtOH, OleT_LA_ exhibits only small fluctuations (less than 3 Å of RMSF) at residues 70 to 80 and 170 to 184 (FG-loop region) (Fig. 8*B*). In contrast, the RMSF values of OleT_JE_ exhibit high fluctuations (greater than a 6 Å increase) (Fig. 8*B*). This high fluctuation may lead to instability and result in a loss of enzyme activity in the presence of EtOH. From the results, both values of RMSD and RMSF clearly indicate that OleT_LA_ is more stable in EtOH than OleT_JE_.

### Dynamics of OleT_LA_ and site-directed mutagenesis studies

I178 in the FG-loop of OleT_LA_ (equivalent to L176 in OleT_JE_) maintains a shorter distance to the C16 atom of C20FA than L176 in OleT_JE_ (Fig. 7, *D* and *E*), implying that I178 may provide substrate interactions at the C16 atom and promote greater decarboxylation activity toward C16FA in OleT_LA_. To further investigate this issue, we analyzed MD simulations data to examine the interactions of I178 (OleT_LA_) and L176 (OleT_JE_) with C16 atom of C20FA, and the dynamics of the FG-loop influencing substrate binding and decarboxylation activity.

The RMSF values revealed that the FG-loop region of OleT_LA_ exhibits greater fluctuation than that of OleT_JE_ (Fig. 8, *C* and *D*). These findings were consistent with the B-factor value which also confirmed the higher fluctuation of the FG-loop of OleT_LA_ (Fig. S14). Although the FG-loop of OleT_LA_ can be flexible, a distance between the C_α_ of I178 in OleT_LA_ and C16 atom of C20FA was stable at around 5.7 Å throughout the 40 ns simulation period. In contrast, the distance between the C_α_ of L176 in OleT_JE_ and C16 atom of C20FA began at 6.5 Å, increased to 15.7 Å after 28 ns, and remained around 15 Å throughout rest of the simulation period (40 ns) (Fig. 8*E*). These findings indicate that I178 in OleT_LA_ can maintain and stabilize substrate within the active site, whereas L176 in OleT_JE_ does not hold the substrate at the optimum binding position.

To explore the functional role of I178 of OleT_LA_ (equivalent to L176 of OleT_JE_) in facilitating C16FA decarboxylation in OleT_LA_, the I178L variant of OleT_LA_ (OleT_LA_ I178L) and the L176I variant of OleT_JE_ (OleT_JE_ L176I) were constructed, and their activities were measured using fatty acids as substrates. The results revealed that OleT_LA_ I178L still maintained and even exhibited increased decarboxylation activity toward C16FA in 2.5% (v/v) of EtOH and toward C14FA, C16FA, and C18FA in 10% (v/v) of EtOH compared to those of the wild-type OleT_LA_ and OleT_JE_ (Fig. 5*D*). From steady-state kinetics data in 10% (v/v) of EtOH, the *k*_cat_ of OleT_LA_ I178L decarboxyation activity was higher than that of wild-type OleT_LA_ (1.1-fold), with no significant difference in the values of *k*_cat_/*K*_M_ (Table 1). The data indicate that having either isoleucine or leucine at position 178 of OleT_LA_ still allows OleT_LA_ to catalyze decarboxylation well. In contrast, the OleT_JE_ L176I variant exhibited a significant loss of decarboxylation activity, with less than 1% decarboxylation activity for C10FA, C12FA, and C14FA, and less than 5% for C16FA and C18FA compared to that of OleT_JE_ wild type. For C20FA, the decarboxylation activity of OleT_JE_ L176I was 37% in 2.5% (v/v) EtOH and 23% in 10% (v/v) EtOH, respectively, compared to that of the wild-type enzyme. Therefore, MD simulations and mutagenesis data both reveal significant differences in the FG-loop dynamics of OleT_LA_ and OleT_JE_. A higher flexibility of the FG-loop in OleT_LA_ than that of OleT_JE_ possibly causes OleT_LA_ to be more mutation-tolerant than OleT_JE,_ especially in the FG-loop region.

We further analyzed product distribution from the reactions of OleT_LA_, OleT_JE_, and their variants using C14FA as substrate (Fig. 5*E*). The results showed that the percentage of decarboxylation product from the reaction of the I178L OleT_LA_ increased compared to that of wild-type OleT_LA_. On the contrary, L176I of OleT_JE_ exhibited a decreased decarboxylation to hydroxylation ratio with significant loss of both activities. This is in agreement with the previous study reporting the importance of L176 in governing the control of decarboxylation and hydroxylation of OleT_JE_, where the L167G variant decreased decarboxylation and increased hydroxylation (30). Mutagenesis at the same position was also reported for the reaction of OleT_SA_ from *Staphylococcus aureus* in which the I177L variant exhibited improved decarboxylation activity for octanoic acid (C8FA), C10FA, and C12FA (20). Altogether, the data suggest that the presence of leucine at this position promotes greater decarboxylation.

### Effects of EtOH on substrate binding and heme state of OleT_LA_

**Figure 9:**
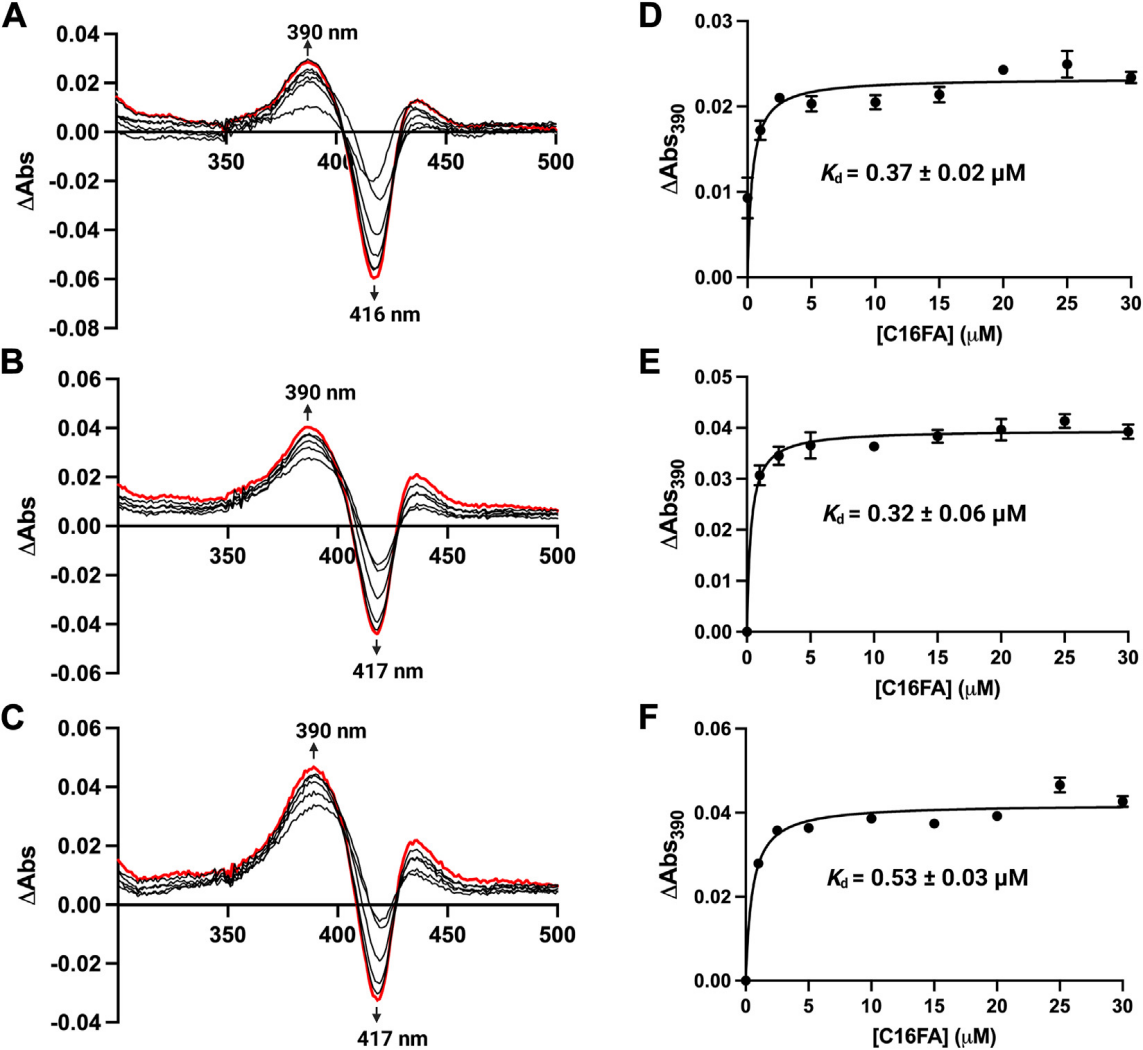
**Heme spectral changes in OleT_LA_ upon C16FA binding in the presence and absence of EtOH.***A,* difference absorbance spectra of OleT_LA_ (5 μM) upon addition of C16FA into a buffer system. *B,* difference absorbance spectra of OleT_LA_ (5 μM) upon addition of C16FA into a 2.5% (v/v) EtOH system. *C,* difference absorbance spectra of OleT_LA_ (2.5 μM) upon addition of C16FA into a 10% (v/v) EtOH system. *Black line*s indicate spectra upon titration of substrate, *red lines* indicate spectra at the maximum C16FA concentration (30 μM). *D–F,* plots of different absorbance at 390 nm values *versus* C16FA concentrations in the systems of buffer, 2.5% (v/v), and 10% (v/v) of EtOH, respectively, were used for calculating *K*_d_ values. Results are shown as the mean ± SD (n = 3).

As the steady-state kinetics data indicated that the reaction of OleT_LA_ was significantly faster in the presence of EtOH, particular at 10% (v/v) EtOH, we thus investigated the effects of EtOH on the heme spectral characteristics of OleT_LA_ upon binding of a fatty acid substrate. Upon substrate binding, the heme spectrum changes from the low spin state (LS) in which water is bound as the sixth ligand to the high spin state (HS) with the fatty acid bound instead (19, 36) (Fig. 9, *A–C*). For OleT_JE_, a previous report showed a spectral shift from LS with λ_max_ at 422 nm to HS with λ_max_ at 390 nm upon the C20FA binding (19). We thus investigated the effects of EtOH (using three different EtOH concentrations: 0%, 2.5%, and 10% (v/v)) on the heme spectral changes upon the binding of C16FA to OleT_LA_. In all systems, the spectrum of the substrate-free OleT_LA_ shifted from the LS state at around 416 to 417 nm to the HS state at 390 nm after addition of C16FA (Fig. 9, *A–C*), indicating that C16FA binding to the enzyme exhibits similar effects with other CYP152s. We also calculated *K*_d_ values for the binding of C16FA to OleT_LA_ from plots of absorbance changes at 390 nm *versus* C16FA concentrations. The *K*_d_ was calculated as 0.37 μM in buffer (0% (v/v) EtOH), 0.32 μM in 2.5% (v/v) EtOH, and 0.53 μM in 10% (v/v) EtOH systems (Fig. 9, *D–F*) which were in agreement with *K*_d_ values obtained from ITC experiments (Table 2).

### Stopped-flow experiments to investigate kinetics of Compound I formation in the OleT_LA_ reaction *via* an H_2_O_2_ shunt in the presence and absence of EtOH

**Figure 10:**
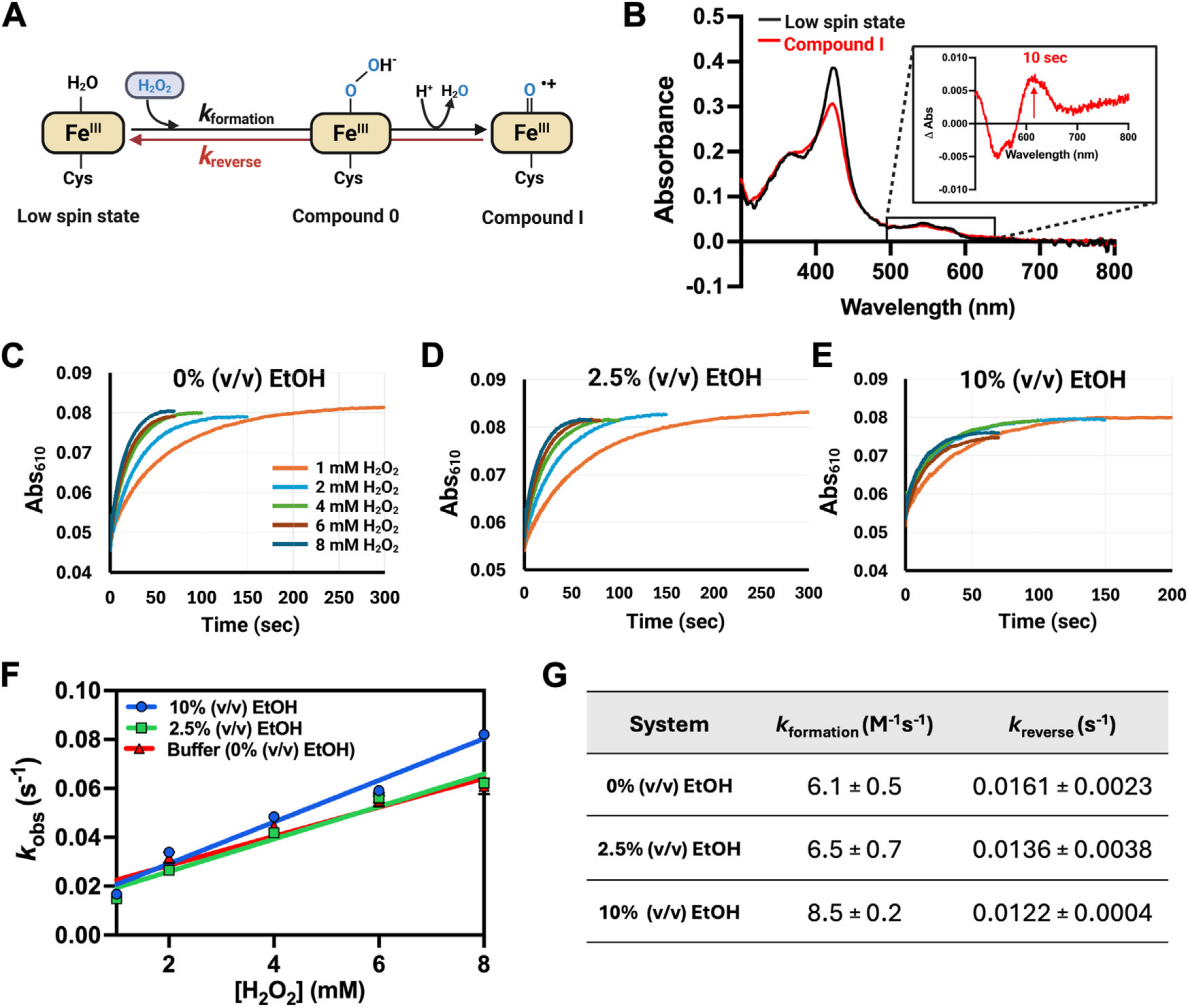
**Transient kinetics of H_2_O_2_-dependent oxidation of OleT_LA_ in three solvent systems.***A*, the mechanism of Compound I formation resulting from the reactions of OleT_LA_ and H_2_O_2_ in the absence of substrate. *B,* absorption spectra of OleT_LA_. The *black line* represents a spectrum of 8 μM of OleT_LA_ before H_2_O_2_ addition while the *red line* represents the enzyme spectrum after adding 1 mM of H_2_O_2_ for 10 s. The inset in B shows the difference absorption of OleT_LA_ after mixing with 1 mM of H_2_O_2_ for 10 s (*red line*). An *arrow* indicates the increase of absorbance at 610 nm after mixing for 10 s. *C–E*, kinetics traces of Abs_610_ increase after mixing OleT_LA_ (8 μM) with H_2_O_2_ (1–8 mM) in the reactions performed in 0%, 2.5%, and 10% (v/v) of EtOH, respectively. Data measured at 610 nm are correlated with the formation of Compound I. *F*, data of *k*_obs_*versus* concentrations of H_2_O_2_ were fitted with a linear function to yield *k*_formation_ (slope) and *k*_*r*everse_ (y-intercept) of Compound I formation. *G,* the table shows the transient kinetic parameters of Compound I formation under three different conditions.

To identify steps in which EtOH may affect the kinetics of intermediate formation in the reaction of OleT_LA_, we carried out stopped-flow experiments to monitor the kinetics of Compound I formation *via* the H_2_O_2_ reaction path (peroxygenase activity, Fig. 10*A*) in the presence and absence of EtOH. In general, various P450 enzymes can react with peroxide compounds such as hydrogen peroxide or meta-chloroperoxybenzoic acid to form Compound 0 and then Compound I. However, the conversion of Compound 0 to Compound I is generally fast, and Compound I is a common species detected along this path. Therefore, we aimed to investigate the effects of EtOH on the kinetics of Compound I formation in OleT_LA_ by carrying out the reactions in the absence of substrate.

A solution of OleT_LA_ (8 μM) was mixed with H_2_O_2_ solutions (1–8 mM) and followed using stopped-flow spectrophotometry. Under these conditions, the reaction proceeded only through the formation of Compound 0, which is rapidly converted to Compound I (Fig. 10*B*) as observed by the increase of absorbance signals at 610 nm, consistent with the previous report for Compound I formation in the reaction of CYP119 (55). We carried out the reactions under three different conditions (0% (v/v) EtOH, 2.5% (v/v) EtOH, and 10% (v/v) EtOH). The data revealed that the reactions containing 10% (v/v) EtOH gave higher *k*_obs_ values of Compound I formation compared to the reactions containing 0% or 2.5% (v/v) EtOH under the same concentrations of H_2_O_2_. The *k*_obs_ values of Compound I formation are linearly dependent on H_2_O_2_ concentrations from 1 mM to 8 mM, indicating that the overall formation of Compound I is mainly controlled by a bi-molecular reaction of H_2_O_2_ reacting with OleT_LA_ (the slowest step) and that the conversion of Compound 0 to Compound I is very rapid (Fig. 10, *C–E*). Plots between *k*_obs_ and H_2_O_2_ concentrations of the three reaction systems are shown in Figure 10*F*. Values of *k*_formation_ and *k*_reverse_ can be calculated from the plots in Figure 10*F,* and the values are summarized in Figure 10*G*. These transient kinetic results clearly indicate that the presence of EtOH facilitates the reaction of H_2_O_2_ with OleT_LA_ to form Compound I.

### Identification of a new redox partner system for CYP152 decarboxylases

**Figure 11:**
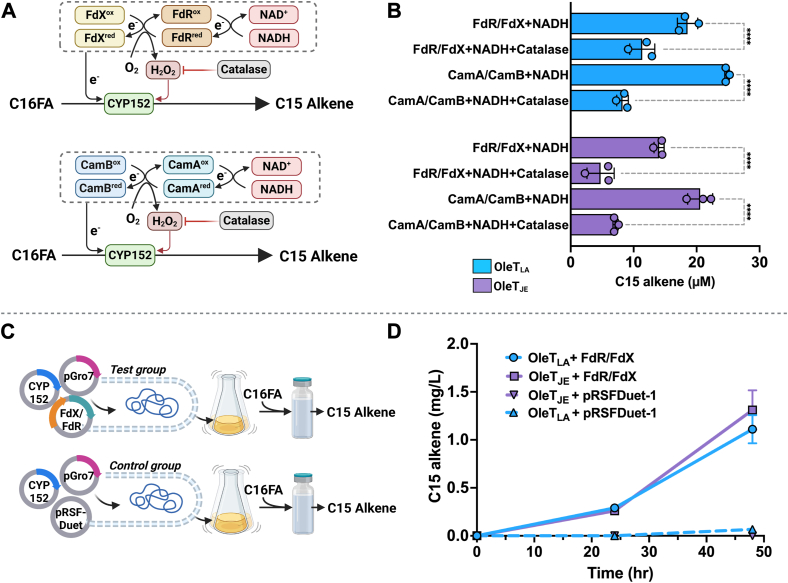
**Multiple turnover reactions of OleT_LA_ and OleT_JE_ using different reductant systems.***A*, the overall scheme explaining possible paths of electron transfer. Addition of catalase (*red line*) can remove H_2_O_2_ resulted from the uncoupling path of redox partner systems. The above *panel* is the FdR/FdX system and the below *panel* is the CamA/CamB system. *B,* products generated from multiple turnover reactions of OleT_LA_ and OleT_JE_ and C16FA using different redox partner systems in the absence and presence of catalase. *C,* the experimental workflow designed to test the ability of FdR/FdX to serve as a redox partner of CYP152 enzymes *in vivo*. *D,* bioconversion of cells overexpressing pGro7, FdR/FdX and OleT_LA_ or OleT_JE_ (test group) compared to those of cells only overexpressing pGro7, OleT_LA_ or OleT_JE_ without FdR/FdX (control group). Details of the experiments are described in Experimental Procedures. Results are shown as the mean ± SD (n = 3). *Asterisks* denote statistical significances (∗∗∗∗*p* ≤ 0.001) determined by two-way ANOVA followed by multiple comparison tests.

To identify new redox partners for CYP152 decarboxylases, we tested the abilities of OleT_LA_ and OleT_JE_ to use ferredoxin reductase/ferredoxin (FdR/FdX) and NADH and compared it to the known redox partner system for CYP152 decarboxylases, putidaredoxin reductase/putidaredoxin (CamA/CamB) and NADH. The overall reaction scheme is shown in Figure 11*A*, and the abilities of both OleT_LA_ and OleT_JE_ and each redox partner system to generate C15 alkene from C16FA are demonstrated in Figure 11*B*. The results indicate that similar to CamA/CamB, FdR/FdX can serve as a redox partner system for both OleT_LA_ and OleT_JE_ for the decarboxylation activity of C16FA. For both redox partner systems, the addition of catalase into the reactions of OleT_LA_ and OleT_JE_ decreased their activities. This was due to removal of H_2_O_2_ resulting from the uncoupling reaction of NADH and FdR or CamA, which was used as a co-substrate *via* the H_2_O_2_ shunt pathway. These results are consistent with the previous study showing that adding catalase in the OleT_JE_ reaction with a redox partner system diminished decarboxylation activities (37, 52). Both CamA/CamB and FdR/FdX systems gave similar amounts of C15 alkene formation in the presence and absence of catalase, indicating that both redox partner systems can be used to provide electrons to both CYP152 enzymes with similar efficiency.

Additionally, we also confirmed that the heme cofactor of both CYP152 enzymes could indeed receive electrons from FdR/FdX and CamA/CamB by observing spectral changes from the ferric state to ferrous state under anaerobic conditions. The results showed that both CamA/CamB and FdR/FdX systems can transfer electrons from NADH to the heme cofactor of both enzymes, resulting in a spectral shift from 528 to 554 nm for OleT_LA_ (Fig. S15, *A* and *B*) and 528 to 564 nm for OleT_JE_ upon the change from ferric to ferrous states (Fig. S16, *C* and *D*). The same patterns could also be obtained upon chemical reduction of the heme cofactor by adding sodium dithionite to OleT_LA_ or OleT_JE_ (Fig. S15, *E* and *F*). We further investigated the stability of the reductase enzyme by measuring its *T*_m_ values. The results measured the *T*_m_ of FdR as 60 °C compared to *T*_m_ of 50 °C for CamA (Fig. S16, *A* and *B*). The higher *T*_m_ of FdR suggests its potential use under more demanding or industrial conditions.

We further demonstrated the use of a redox partner system for *in vivo* bioconversion using *Escherichia coli* BL21 (DE3) cells co-transformed with pGro7, FdR/FdX system, and OleT_LA_ or OleT_JE_. Cellular NAD(P)H serves as a reductant in these experiments, and the experimental design is shown in Figure 11*C*. For the bioconversion, 128.22 mg/L of C16FA was added at the starting time. The results showed that 0.42 mg/L of C15 alkene could be produced after 24 h and increased to around 1.26 mg/L after 48 h in both cells overexpressing OleT_LA_ and OleT_JE_ (Fig. 11*D*). The C15 alkene product was not detected in the control system with no redox partners. These results indicate that the new redox partner system FdR/FdX identified in this work can be used for both *in vitro* and *in vivo* conversion.

## Discussion

Our work reports on the biophysical and catalytic properties of a new CYP152 fatty acid decarboxylase from *L. alkaliphilus* (OleT_LA_), which can catalyze the decarboxylation of C16FA in the presence of 10% EtOH with greater activity than OleT_JE_, the well-studied CYP152 enzyme of the same class. In-depth and mechanistic investigations of OleT_LA_ revealed its structural and dynamic features that are distinctively different from those of OleT_JE_. OleT_LA_ has a spacious active site pocket which accomodates a flipped-heme configuration and large numbers of hydrophobic clusters, which may attribute to the enzyme’s tolerance to 10% (v/v) EtOH. Molecular dynamics simulations have also revealed that various regions of OleT_LA_ can maintain their backbone conformation in the presence of EtOH better than those of OleT_JE._ Site-directed mutagenesis of a key substrate-interacting residue in OleT_LA_ yielded an active variant equivalent to the wild-type enzyme, while the similar mutation of OleT_JE_ resulted in near abolishment of enzyme activity. These data imply that the OleT_LA_ structural scaffold tolerates mutations better than that of OleT_JE_. Investigation of the kinetics of Compound I formation by OleT_LA_ indicates that Compound I forms faster in the presence of EtOH, explaining the root cause of activity enhancement of OleT_LA_ in the presence of 10% (v/v) of EtOH. In addition, an improved method for expression and purification, and identification of new redox partners of CYP152 enzymes, which should be useful for studying CYP152 enzymes in general, are also reported in this work.

The X-ray structure of OleT_LA_ revealed a flipped heme configuration that differs from almost all other CYP152s. The active site cavity analysis using CavityPlus (56) showed that the cavity volume of the active site of OleT_LA_ is larger than that of OleT_JE_ by around 2.3-fold. We hypothesize that a large active site cavity is a factor accommodating the flipped heme configuration of OleT_LA_. Additionally, the spacious cavity may enable greater substrate flexibility within the active site, potentially leading to increased hydroxylation activity compared to OleT_JE_. A similar correlation between enhanced substrate dynamics and increased hydroxylation has been reported in P450_BSβ_ (57).

The reactions of OleT_LA_ could be enhanced in the presence of 10% EtOH. A previous study reported that, in co-solvent systems, CYP152K6 and its Q87H variant did not show an increase in the total turnover number (TTN) when their reactions were performed in 3% (v/v) acetone and 0.01% (v/v) Triton X-100. In contrast, OleT_JE_ demonstrated an increased TTN in 5% (v/v) acetone when using C12FA, C16FA, and C18FA as substrates (41). In this study, we explored the use of EtOH as a co-solvent in the catalytic system and found that OleT_LA_ exhibited higher catalytic efficiency and turnover for C16FA decarboxylation compared to OleT_JE_ in the presence of 10% (v/v) EtOH (Table 2). Moreover, 10% (v/v) EtOH significantly improved the TTN of OleT_LA_ when using C12FA, C14FA, and C16FA as substrates, compared to reactions performed in 2.5% (v/v) EtOH (Fig. S6).

To identify the underlying effects of EtOH on OleT_LA_, we performed MD simulations in the presence and absence of EtOH. RMSD values revealed that the overall structure of OleT_LA_ maintained its conformation in the presence of EtOH more stably compared to OleT_JE_. Additionally, RMSF analysis showed that the FG-loop of OleT_LA_ exhibited greater flexibility than that of OleT_JE_, consistent with the B-factor values, which also indicate high flexibility of the FG-loop region in OleT_LA_ (Fig. S14, *A*–*C*). The ITC data further suggested that the binding of C16FA to OleT_LA_ is entropy-driven, likely due to hydrophobic interactions or conformational changes upon substrate binding (58). Notably, the addition of 10% (v/v) EtOH enhanced the entropy-driven nature of substrate binding, indicating that EtOH influences the conformational dynamics during the binding process. However, C16FA titration showed no significant difference in binding affinity in the presence or absence of EtOH.

To investigate the effects of EtOH on catalysis, we performed transient kinetic analyses of Compound I formation in OleT_LA_ under conditions both with and without EtOH. Although transient kinetics have been previously studied in CYP152 enzymes, those studies primarily focused on the decay of Compound I and the formation of Compound II to differentiate between decarboxylation and hydroxylation mechanisms (30, 59). To date, the kinetics of Compound I formation by hydrogen peroxide in the absence of a substrate have not been reported for CYP152s. In earlier studies, OleT_JE_ and OleT_SA_ exhibited a characteristic absorption peak for Compound I at 690 nm (59, 60, 61, 62). However, our results show that, in the absence of substrate, Compound I exhibits a characteristic spectrum at 610 nm, similar to the results reported for CYP119 (55). Our findings indicate that the *k*_obs_ of Compound I formation is linearly dependent on H_2_O_2_ concentration. Interestingly, the presence of 10% EtOH resulted in a higher rate constant of Compound I formation compared to the condition without EtOH. Altogether, our results confirm that EtOH not only improves substrate solubility but also affects dynamics of OleT_LA_, promotes entropy-driven substrate binding, and increases the rate of Compound I formation.

OleT_LA_ exhibits greater FG-loop flexibility than OleT_JE_, which may influence the substrate tunnel to orient substrate binding in this region. Despite flexibility in the FG-loop region, C_α_ of I178 can still maintain interactions with C16 atom of C20FA which may contribute to higher decarboxylation activity of OleT_LA_ than that of OleT_JE_. Interestingly, the I178L variant of OleT_LA_ showed enhanced decarboxylation activity compared to its wild-type enzyme. Conversely, introducing the reciprocal L176I mutation into OleT_JE_ led to a significant loss of decarboxylation activity. This indicates that this region of OleT_LA_ is more tolerance to mutation than OleT_JE_, possibly due to its greater flexibility. In general, protein mutations frequently occur in disordered or flexible loop regions due to their structural tolerance to variation (63, 64). Therefore, we hypothesize that the dynamics within the F-G loop of OleT_LA_ allow it to better tolerate mutations.

The co-expression systems of CYP152 with *Gro*EL and *Gro*ES to improve soluble expression and with ferrochelatase to catalyze the insertion of ferrous iron into protoporphyrin IX to synthesize a functional heme *in vivo* (31) could improve holoenzyme expression. Adding δ-ALA and hemin to the culture medium can increase the heme content. The results indicated that δ-ALA and heme synthesis in *E. coli* BL21 (DE3) are not sufficient, which is in agreement with the previously reported results (65). Our data reported in Table S1 also indicate that the combined methods (P2) with hemin addition could provide the highest levels of holoenzyme production among CYP152 enzymes. We envision that the method reported here should be useful for the overexpression of other P450 enzymes in general.

In conclusion, our findings report significant advancements in understanding the biochemical, biophysical, and structural features important for controlling decarboxylation activities in CYP152 fatty acid decarboxylases. Unique structural dynamics of OleT_LA_ allow it to tolerate EtOH and mutation better than its homolog enzyme. Collectively, all data reported here provide valuable insights into the structural and functional properties of CYP152s, which are an important foundation for future enzyme engineering for industrial applications in the future.

## Experimental procedures

### Materials

All chemicals and solvents used were commercially available and high purity, analytical or HPLC grade. Standard compounds of fatty acids and alkenes were purchased from Tokyo Chemical Industry (TCI). Standard compounds of 2-hydroxy tetradecanoic acid, 3-hydroxy tetradecanoic acid, 2-hydroxy hexadecanoic acid, and 3-hydroxy hexadecanoic acid were purchased from Sigma Aldrich. Hemin, ammonium iron (III) citrate, imidazole, polyethylenimine, and reduced form of nicotinamide adenine dinucleotide (NADH), and catalase from bovine liver were purchased from Sigma Aldrich. Peptone and yeast extract were purchased from Himedia.

δ-Aminolevulinic acid (δ-ALA) was purchased from Formedium. Ethyl acetate was purchased from Honeywell. Sodium dihydrogen phosphate (NaH_2_PO_4_) was purchased from Merck. The N-2-hydroxyethylpiperazine-N-2-ethane sulfonic acid (HEPES) and sodium chloride (NaCl) were purchased from Glentham LIFE SCIENCES. Protein markers and reagents for SDS-PAGE analysis and protein markers were purchased from Enzmart, Thailand.

### Analysis of sequence similarity network (SSN)

A sequence similarity network (SSN) was constructed using the Enzyme Function Initiative-Enzyme Similarity Tool (EFI-EST) (57, 66). The UniProt database was used to retrieve sequences with 60 to 100% identity to OleT_JE_. These sequences were used to generate a sequence similarity network (SSN) of OleT_JE_ and its homologs. The SSN was visualized as a full network using Cytoscape version 3.2.1 (58, 67).

### Co-overexpression of CYP152 with different accessory plasmids

All genes in this experiment were synthesized by Genscript, and a pGro7 plasmid was purchased from Takara Bio. Details of genes, vectors, and antibiotic markers used for each gene selection are summarized in Table S3.

*E. coli* BL21 (DE3) containing the pGro7 plasmid (*Gro*EL and *Gro*ES) was prepared as a competent cell for transformation of the OleT_LA_ plasmid or other accessory plasmids. The transformants were incubated in LB broth at 37 °C, 220 rpm for 1.5 h, then spread on LB agar with an appropriate antibiotic and incubated at 37 °C for 12 to 16 h. A single colony was picked to start a culture in an auto-induction medium with appropriate antibiotics. The culture was incubated at 37 °C, 220 rpm for 16 h to use as a cell starter.

### Enzyme expression system for enhancing the production of holoenzyme

The starter culture described above was inoculated into 650 ml of auto-induction medium with 1% (v/v) in the presence of ammonium iron (III) citrate (20 μg/ml), l-arabinose (2 mg/ml) to induce expression of *Gro*EL and *Gro*ES genes. Ampicillin (50 μg/ml), kanamycin (34 μg/ml), or chloramphenicol (20 μg/ml) resistant genes were used for plasmid selection as summarized in Table S3. The different expression systems were separated into 7 groups with different accessory plasmids and supplement compounds (Fig. 2*A*). The cultures were grown at 37 °C until OD_600_ reached about 0.8 to 1.0 and then further grown at 25 °C for 20 h. The cell culture was harvested by centrifugation and kept at −80 °C until use.

### Hemin addition effects on holoenzyme production

For purification of CYP152, the cell pellet was resuspended in 50 mM NaH_2_PO_4_, 300 mM NaCl and 20 mM imidazole at pH 8.0 and lysed on ice using ultrasonication. After centrifugation, 0.1% (v/v) polyethylene amine (PEI) was added to the supernatant and centrifuged to collect a clear supernatant for further steps. Three purification protocols were tested. *Method 1* (P1) loaded the supernatant directly onto a nickel nitriloacetic acid (Ni-NTA) affinity chromatography column without hemin incubation. *Method 2* (P2) added hemin (0.02 mg/1 mg of total protein) to the supernatant and followed by incubation for 16 h. Then, the supernatant was centrifuged, and the precipitate was removed before loading the solution onto a Ni-NTA affinity chromatography column, and Sephadex G-25 column. Extra hemin was removed from the supernatant solutions by the 2 columns mentioned above. For *Method 3* (P3), hemin (0.02 mg/1 mg of total protein) was added to the purified enzyme obtained from P2 and incubated for 16 h. The solution was then centrifugated to remove any precipitate and the clear supernatant was loaded onto a Sephadex G-25 column to remove extra hemin. For all methods, CYP152 was purified using Ni-NTA affinity chromatography column by washing and eluting the column with buffers containing 50 mM and 250 mM imidazole, respectively. Imidazole was removed from the purified enzyme and exchanged into a storage buffer of 50 mM NaH_2_PO_4_, 300 mM NaCl, pH 7.5 by passing through a Sephadex G-25 column. The purified protein solution from each step was analyzed by SDS-PAGE. To evaluate the ratio of holoenzyme to total protein, Bradford and pyridine hemochromagen assays (40) were used to measure the amount of total purified enzyme and total purified holoenzyme, respectively. The total purified holoenzyme was calculated based on pyridine hemochromagen assays and presented per 1 L of culture medium.

### Optimization of expression and purification systems

*E. coli* BL21 (DE3) containing a pGro7 plasmid (*Gro*EL and *Gro*ES) was used as a competent host for transformation. The OleT_LA_ or OleT_JE_ plasmid was co-transformed into the *E. coli* BL21 (DE3) competent cells with FC gene. The transformants were incubated in LB broth at 37 °C, 220 rpm for 1.5 h, and then spread on an LB agar with ampicillin (50 μg/ml), kanamycin (34 μg/ml), and chloramphenicol (20 μg/ml) and incubated at 37 °C for 12 to 16 h. A single colony was picked for inoculating into a starter culture and 1% (v/v) of the starter was then inoculated into 650 ml of auto-induction medium with ammonium iron (III) citrate (20 ug/ml), l-arabinose (2 mg/ml), antibiotic drugs including ampicillin (50 μg/ml), kanamycin (34 μg/ml), and chloramphenicol (20 μg/ml). The culture medium was supplemented with hemin (0.05 mM) and δ-ALA (0.5 mM), grown at 37 °C, 220 rpm until OD_600_ reached about 0.8 to 1.0, and then grown at 25 °C for 20 h before being harvested by centrifugation.

For purification steps, the cell pellet was resuspended in 50 mM NaH_2_PO_4_, 300 mM NaCl, and 20 mM imidazole at pH 8.0 and lysed on ice using ultrasonication. After centrifugation, the supernatant was collected, added 0.1% (v/v) PEI, centrifuged to discard the pellet, and the supernatant was collected for use in further steps. The supernatant was added to the hemin (0.02 mg/1 mg of total protein) and incubated for 16 h. The supernatant was then centrifuged before loading it onto a Ni-NTA affinity chromatography column. The column was washed and eluted with buffers containing 50 mM and 250 mM imidazole, respectively. Extra imidazole was next removed by passing an enzyme solution through a Sephadex G-25 gel filtration column. The extra hemin was removed during Ni-NTA affinity chromatography column and also afterward by passing through a Sephadex G-25 gel filtration column. The purified enzyme was stored in 50 mM NaH_2_PO_4_, 300 mM NaCl, pH 7.5 (storage buffer) at −80 °C. The enzyme purity was checked by SDS-PAGE.

The methods used for expression and purification of redox partner enzymes, including FdR, FdX, CamA, and CamB, are described in the Supplementary Methods.

### Sample preparation and GC-MS analysis

A reaction was typically quenched by adding 2-fold reaction volume of ethyl acetate which contained 30 μM of tetradecane as an internal standard. The reaction mixtures were vortexed for 1 min and then centrifuged at 12,000 rpm, 4 °C for 10 min to separate an organic and an aqueous phase. The organic phase was injected into an Agilent gas chromatography system (GC-MS) (Agilent 7890B equipped with Agilent 5977B MSD) equipped with a HP5-MS column (30 m × 0.25 mm × 0.25 μM) (Agilent), and helium was used as a carrier gas. The oven conditions for C10FA and 1-nonene (C9 alkene) were ramped up from 60 to 240 °C with a rate of 19 ° C min^−1^ and held at 240 °C for 1 min. For C12FA, 1-undecene (C11 alkene), C14FA, 1-tritradecene (C13 alkene), C16FA, and 1-pentadecene (C15 alkene), the oven was ramped up from 60 to 250 °C with a rate of 12 °C min^−1^ and hold at 250 °C for 4 min. For C18FA and 1-heptadecene (C17 alkene), the oven was ramped up from 60 to 250 °C with a rate of 22 °C min^−1^ and hold at 250 °C for 4 min. For C20FA and 1-nonadecene (C19 alkene), the oven was ramped up from 100 to 250 °C with a rate of 23 °C min^−1^ and held at 250 °C for 6 min. The response of target compounds was normalized with signals of the internal standard. The concentration of each compound was calculated based on a standard calibration curve.

### Sample preparation and HPLC-MS analysis

For analyzing hydroxylated products of fatty acids, the reactions were quenched by adding an equal volume of acetonitrile, centrifuged, and filtrated. The analytes (10 μl) were injected into a liquid chromatography coupled with diode array and single quadrupole mass spectroscopy detection (LC-DAD/MS) (Agilent 1290 Infinity II LC System equipped with DAD, Single Quadrupole Mass Spectrometers system). A Zorbax eclipse plus C18 column (5 μm, 4.6 × 250 mm) (Agilent Technology) was used for separation. The mobile phase system A was water with 0.1% (v/v) formic acid, and system B was acetonitrile with 0.1% (v/v) formic acid. The separation was done using isocratic solvents of 60% B and 40% A, 70% B and 30% A, 80% B and 20% A, 90% B and 10% A. 98% B and 2% A, and 100% A and 0% B for analyzing hydroxylated products of C10FA, C12FA, C14FA, C16FA, C18FA and C20FA, respectively. Single hydroxylation products of C10FA, C12FA, C14FA, C16FA, C18FA, and C20FA were monitored using selected ion monitoring systems with a negative mode and extracted mass of 187, 215, 243, 271, 299, and 328 m/z, respectively.

### Investigation of enzyme thermostability

Thermofluor assays, which report intensity of SYPRO Orange Protein Gel Stain (Thermo Fisher Scientific Inc.) at different temperatures in the presence of OleT_LA_ or OleT_JE,_ were used for measuring the enzyme melting temperatures because fluorescence dye is drastically enhanced when the protein is denatured. The reactions containing 10 μM of enzyme, 25X of SYPRO Orange Protein Gel Stain solution in 25 μl of a storage buffer were carried out in a CFX96 Real-Time (Bio-Red) Thermal Cycle. The temperature of the experiments was raised from 30 to 80 °C at a continuous rate of 1 °C min^−1^. The temperature at which half of the total amount of protein transitioned into an unfolded form is designated as the melting temperature.

We also further investigated thermostability of each enzyme by preincubating the enzyme at two different temperatures at 30 °C and 45 °C and at various incubation times. Residual activity at each time point was determined by the activity assay which used 200 μM of C14FA as a substrate, and 400 μM of H_2_O_2_ as co-substrate. Reactions were set at 25 °C for 1 h, and the amount of C13 alkene generated was determined using GC-MS after being quenched and extracted with 2-fold volume of ethyl acetate.

### Reaction optimization

To optimize the conditions for bioconversion experiments, we first explored reactions of OleT_LA_ and OleT_JE_ at various H_2_O_2_ concentrations, pHs and percentage of EtOH as a co-solvent. We first varied the concentration of H_2_O_2_ from 0.2 to 2.0 mM with 200 μM of C14FA in the reaction. The results in Fig. 5*A* indicate that the optimal concentration of H_2_O_2_ to yield the highest C13 alkene was 400 to 600 μM for OleT_LA_ and 400 to 1000 μM for OleT_JE_. Therefore, 400 μM of H_2_O_2_ was used for the reactions of both enzymes to explore the optimum EtOH and pH. We explored the addition of EtOH varying from 2.5 to 20% (v/v) and found that both OleT_LA_ and OleT_JE_ tolerated EtOH up to 10% (v/v) (Fig. 5*B*). For investigating effects of pH, the reactions varied in pH range from 4 to 11 using several buffer types including 50 mM sodium acetate buffer for pH 4 to 6, 50 mM sodium dihydrogen phosphate buffer for pH 7.5, and 50 mM sodium pyrophosphate buffer for pH 9 to 11. All buffers were adjusted ionic strength to the same level (68). All reactions were performed in 50 mM NaH_2_PO_4_ buffer with 300 mM NaCl, pH 7.5, at 25 °C for 1 h, except those investigating the effects of pH, where reactions were performed in a different buffer type and pH at 25 °C for 1 h. The amount of C13 alkene was measured using GC-MS after the reactions were quenched and extracted with two-fold volume of ethyl acetate.

### Analysis of CYP152 activities with different chain lengths of fatty acids using H_2_O_2_ as a co-substrate

Fatty acids, including decanoic acid (C10FA), dodecanoic acid (C12FA), tetradecanoic acid (C14FA), hexadecanoic acid (C16FA), octadecanoic acid (C18FA), and icosanoic acid (C20FA), were tested for their abilities to serve as substrates for OleT_LA_ and OleT_JE_. All fatty acids were prepared in absolute EtOH at 20 mM as stock solutions. The reaction was carried out in 50 mM NaH_2_PO_4_ buffer with 300 mM NaCl, pH 7.5, with 2.5 or 10% (v/v) of EtOH, 200 μM of a substrate, and 400 μM of H_2_O_2_. The reactions were performed at 25 °C for 1 h, and then the alkene product was measured using GC-MS after the reactions were quenched and extracted with two-fold volume of ethyl acetate.

### Steady-state kinetics

Steady-state kinetic parameters of OleT_LA_, OleT_JE,_ and OleT_LA_ I178L reactions were determined from initial rates of the reactions performed in 50 mM NaH_2_PO_4_, 300 mM NaCl pH 7.5, containing 2.5% (v/v) or 10% (v/v), 0.3 μM of each enzyme, 400 μM of H_2_O_2_, and C16FA at concentrations ranging between 5 to 250 μM in a final volume of 1 ml at 25 °C. Under specific times between 0.1 to 2 min, the reaction samples were taken and quenched by adding an equal volume of ethyl acetate. The ethyl acetate layer would contain the C15 alkene product, which was measured using GC-MS. Values of initial rates were plotted against substrate concentrations to analyze for *K*_m_ and *V*_max_ values using the Michaelis–Menten equation by GraphPad Prism software version 10.

### Investigation of substrate binding using isothermal titration calorimetry (ITC)

The binding reactions contained 100 μM of OleT_LA_ or 200 μM of OleT_JE_ and 10 μM of C16FA were monitored heat changes using MicroCal PEAQ-ITC (Malvern Panalytical). Both enzyme and C16FA were dissolved in 50 mM NaH_2_PO_4_ buffer with 300 mM NaCl at pH 7.5 (storage buffer) in the presence of 2.5% (v/v) EtOH or 10% (v/v) EtOH. The enzyme solution was filled into a syringe while the C16FA solution was filled into the ITC cell. The reactions were constantly stirred at 750 rpm while controlling the temperature at 25 °C during titration. A total of 19 enzyme injections were added into the substrate solution. At the first injection, 0.4 μl of the enzyme solution was added, and this data point was omitted from the analysis to eliminate error from diffusion effects. For the other 18 injections, 2 μl of the enzyme solution was added with 4 s of injection spacing and 150 s of injection duration to ensure that the conditions are under equilibrium. The data were processed and analyzed using the Microcal PEAQ-ITC analysis software to calculate the *K*_d_ value based on a single-site binding model.

### Crystallography of OleT_LA_

Crystals of OleT_LA_: C20FA complex were obtained by the hanging drop vapor diffusion method at 293 K. Before setting up the hanging drop crystallization, a solution of 20 mg/ml OleT_LA_ was incubated with 1 mM of C20FA at 293 K for 1 h. The hanging drop crystallization was then prepared by mixing equal volumes of a protein solution and a reservoir solution containing 3.5 M sodium formate, 0.1 M sodium chloride, and 0.1 M hydroxyethyl piperazineethanesulfonic acid (HEPES), pH 7.5. For X-ray intensity data collection, single crystals were transferred into a cryo-protectant solution containing the same crystallization buffer with 25% (v/v) glycerol and flash-frozen in liquid nitrogen. Diffraction data were collected on beamline BL44XU at SPring-8 using an EIGER X 16M detector (Dectris) at cryogenic temperature (100 K). The native data set was processed and scaled using XDS (69). Structural phases were solved by molecular replacement using PHASER (70) in the CCP4 suite (71) with CYP152L1 (PDB: 4L54) as a search model. Structural refinement was performed using phenix.refine (72), and COOT (73). The data collection and refinement statistics are shown in Table 3.

### Tunnel analysis

To identify substrate tunnels, the CAVER Web program (74) was used for analyzing the structure files of OleT_JE_ (PDB: 4L40) and OleT_LA_ (PDB: 9JQM). The tunnels were identified using default parameters, including minimum probe radius: 0.9; shell depth: 4; shell radius: 3; clustering threshold: 3.5; maximal distance: 3; and desired radius: 5.

### Molecular dynamics simulations

Crystal structures of OleT_JE_ (PDB: 4L40) (36) and OleT_LA_ (PDB: 9JQM) were used for molecular dynamics (MD) simulations. Hydrogen atoms of amino acid residues were added by considering results from the PROPKA software (75). The atom types in the topology files were assigned based on the CHARMM36 parameter set (76). Both structures were solvated in a cubic box of TIP3P water extending at least 15 Å from each direction in the solute. The dimensions of the solvated system was 88 x 91 x 97 Å. MD simulations were carried out using the NAMD program (77) with simulation protocols adapted from previous works (78, 79, 80, 81, 82). The simulations were started by minimizing hydrogen atom positions for 3000 steps followed by water minimization for 6000 steps. The system water was heated to 380 K for 5 ps and then was equilibrated for 15 ps. The whole system was minimized for 10,000 steps and heated to 380 K for 20 ps. After that, the whole system was equilibrated for 180 ps followed by a production stage for 40 ns. The same procedure was applied to both OleT_LA_ and OleT_JE_ systems. The dimensions of the solvated system was 82 x 92 x 102 Å. OleT_LA_ and OleT_JE_ systems were prepared then minimized, heated and equilibrated. The systems were performed MD simulations for 40 ns. The RMSD, RMSF, and some distances of important residues were monitored. Stability comparisons between OleT_JE_ and OleT_LA_ variants were investigated. Distances between the residue pair at 380 K are shown in the results section.

### Site-directed mutagenesis

Site-directed mutagenesis of I178L of OleT_LA_ and L167I of OleT_JE_ was done with overlaping PCR that used OleT_LA_ or OleT_JE_ plasmids as templates (Table S4). The PCR products were treated with *Dpn*I in cut smart buffer at 37 °C for 16 h, transformed into *E. coli* XL−1 blue competent cells and grown at 37 °C on LB agar containing 50 μg/ml ampicillin. The plasmids of selected colonies were extracted for sequence verification. The plasmid containing the correct sequence was transformed into *E. coli* BL21 (DE3) for enzyme expression. The expression and purification methods of variants were the same as those of wild-type OleT_LA_ and OleT_JE_.

### Substrate binding to OleT_LA_

Equilibrium binding of substrate (C16FA) to OleT_LA_ was investigated using spectrophotometry because binding of C16FA to the enzyme showed spectral perturbation with absorbance changes at 390 and 420 nm. Difference spectra of C16FA-bound enzyme at various C16FA concentrations and free enzyme were obtained upon addition of C16FA to an enzyme solution in the main cuvette, whereas the same volume of buffer was added into an enzyme solution in the reference cuvette of a double-beam spectrophotometer. The binding isotherm was calculated from plots of absorbance changes against C16FA concentrations. Data were analyzed using non-linear regression with a single site binding model of GraphPad Prism 10.0 to calculate dissociation constants (*K*_d_) of C16FA binding to OleT_LA_ in different solvent systems including buffer (50 mM NaH_2_PO_4_, 300 mM NaCl, pH 7.5), 2.5% (v/v) of EtOH in buffer and 10% (v/v) in buffer. A stock solution of C16FA (10 mM) was prepared in 80% (v/v) EtOH and 20% (v/v) Triton X-100.

### Rapid kinetics studies using stopped-flow spectrophotometry

Rapid kinetics experiments were conducted using a stopped-flow spectrophotometer (TgK Scientific model SF-61SX). Compound I formation was investigated by mixing 8 μM of OleT_LA_ with H_2_O_2_ (1–8 mM, pseudo-first order conditions). The reactions were performed in 0%, 2.5%, and 10% (v/v) of EtOH in storage buffer (50 mM NaH_2_PO_4_, 300 mM NaCl, pH 7.5). A solution of OleT_LA_ in three different solvent systems as described above was mixed with different concentrations of H_2_O_2_ (1–8 mM). The increase in absorbance at 610 nm indicates Compound I formation (55). Observed rate constants (*k*_obs_) were analyzed using a single exponential function equipped in the Kinetic Studio software. *k*_obs_ values were plotted against H_2_O_2_ concentrations to calculate *k*_formation_ and *k*_reverse_.

### Analysis of electron transfer to the enzyme-bound heme

To observe electron transfer to the enzyme-bound heme using a redox partner system, 5 μM of OleT_LA_ or OleT_JE_, 10 μM of each redox partner system, and 50 μM of C16FA were premixed inside an anaerobic glove box (<0.0004% O_2_, Belle Technology). An enzyme spectrum indicating its ferric form of OleT_LA_ or OleT_JE_ was recorded from 300 to 800 nm. 200 μM of NADH was added to the enzyme solution to start the reaction and observe the change of ferric to ferrous form. The parallel reaction was performed using sodium dithionite as a reducing agent to observe the conversion from ferric to ferrous form.

### Analysis of CYP152 activities using redox partner systems

Multiple turnover reactions of OleT_LA_ and OleT_JE_ using redox partner systems in the presence or absence of catalase were tested (Fig. 11*A*). The reactions contained 5 μM of OleT_LA_ or OleT_JE_, 200 μM of C16FA, 10 μM of each redox partner, and 500 μM of NADH in the presence and absence of 0.2 mg/ml catalase. Reactions were performed in 300 μl of a total volume in 50 mM NaH_2_PO_4_ buffer, pH 7.5, 300 mM NaCl, 2.5% (v/v) EtOH at 25 °C for 1 h. The amount of C13 alkene formed was determined using GC-MS after the reactions were quenched and extracted with two-fold volume of ethyl acetate.

### Bioconversion using redox partner systems

The pRSFDuet−1 vector containing FdR/FdX and OleT_LA_ or OleT_JE_ was transformed into competent *E. coli* BL21 (DE3) containing a pGro7 plasmid. The transformed cells were incubated in LB broth at 37 °C, 220 rpm for 1.5 h, spread on LB agar with ampicillin (50 μg/ml), kanamycin (34 μg/ml), and chloramphenicol (20 μg/ml), and incubated at 37 °C for overnight. A single colony was inoculated into a starter culture in an auto-induction medium with appropriate antibiotics and 1% (w/v) of glucose. The culture was then incubated at 37 °C, 220 rpm overnight. Then, 1% (v/v) of the starter was inoculated into the auto-induction medium in the presence of ammonium iron (III) citrate (20 ug/ml), l-arabinose (2 mg/ml), ampicillin (50 μg/ml), kanamycin (34 μg/ml), and chloramphenicol (20 μg/ml), hemin (0.05 mM) and δ-ALA (0.5 mM) and then grown at 37 °C, 220 rpm until OD_600_ reached about 0.8 to 1.0. Next, 1 ml of cell culture was transferred into 20 ml vial, 128.22 mg/L of C16FA, and shaken at 220 rpm at 25 °C for 24 h and 48 h. At a specific time point, the culture medium was sampled and extracted by adding two-fold ethyl acetate with tetradecane as an internal standard. The mixture was vortexed and centrifuged at 4 °C for 10 min. The organic phase was collected and injected into the GC-MS system.

## Data availability

Data of X-ray structures are available at Protein Data Bank under PDB code 9JQM. The nucleotide sequence of OleT_LA_ is available at National Center for Biotechnology Information (NCBI) under code PQ308727.

## Supporting information

This article contains supporting information (40).

## Conflict of interest

The authors declare that they have no conflicts of interest with the contents of this article.
