# Supplementary material for: Unique structural features define the decarboxylation activity of a CYP152 fatty acid decarboxylase from Lacicoccus alkaliphilus
Source: J Biol Chem. 2025 Jun 19;301(7):110397. doi: 10.1016/j.jbc.2025.110397 (PMC12284513; doi:10.1016/j.jbc.2025.110397)
Supplement: Supplementary Material [file mmc1.pdf]

Unique structural features define the decarboxylation activity of a CYP152 fatty acid decarboxylase from *Lacicoccus alkaliphilus*

Suppalak Phaisan[1], Aisaraphon Phintha[1], Duangthip Trisrivirat[1], Narin Lawan[2], Jeerus Sucharitakul[3], Ailada Charoenpol[1], Pratchaya Watthaisong[1], Hideaki Tanaka[4] , Genji Kurisu[5,6] and Pimchai Chaiyen[1*]

[1]School of Biomolecular Science and Engineering, Vidyasirimedhi Institute of Science and Technology (VISTEC), Wangchan Valley, Rayong, Thailand.

[2]Department of Chemistry, Faculty of Science, Chiang Mai University, Chiang Mai, 50200, Thailand.

[3]Department of Biochemistry, Faculty of Dentistry, Chulalongkorn University, Bangkok, Thailand.

[4]Techno Pro, Inc. TechnoPro R&D Company, 5-5-2, Minatojima-Minamimachi, Chuo-ku, Kobe, Hyogo 650-0047, Japan.

[5]Institute for Protein Research, Osaka University, Suita, Osaka, Japan.

[6]Department of Macromolecular Science, Osaka University, Toyonaka, Osaka, Japan.

[*]Corresponding authors.

E-mail addresses: pimchai.chaiyen@vistec.ac.th

Supplementary methods

Pyridine hemochromagen assays

Determination of heme incorporated into a CYP152 enzyme was carried out using the pyridine hemochromagen assay (1). The assays used two solutions. Solution I contained 0.2 M NaOH, 40% (v/v) pyridine and 500 µM potassium ferricyanide ($K_3[Fe(CN)_6]$). Solution II contained 0.5 M sodium dithionite in 0.5 M NaOH. The analysis was carried out according to the previous reference. First, Solution I was mixed with 50 mM NaH_2PO_4, 300 mM NaCl pH 7.5 (storage buffer) in the cuvette at a 1:1 ratio. The spectrum of this solution was recorded and used as blank. Second, 0.5 ml of Solution I was then mixed with 0.5 ml of purified enzyme which was diluted in the storage buffer in a cuvette (a final absorbance of this solution at 420 nm was around 1). The enzyme solution was recorded its spectrum at 200-800 nm to obtain the oxidized (heme) enzyme spectrum. After that, 10 µl of Solution II was then added to the oxidized enzyme sample and mixed well. At this

stage, the sample should turn into red because the heme cofactor was reduced. The sample spectra should immediately record every minute until the absorbance characteristics do not further change. For calculations, the absorption coefficients of reduced Pyr2-heme b (34.7 mM^{-1} cm^{-1} at 557 nm) and dilution factors were used to calculate the concentration of heme bound to the purified enzyme according to Beer's law (Absorbance = extinction coefficient × concentration × pathlength).

Expression and purification of putidaredoxin reductase (CamA)

E. coli BL21 (DE3) containing a pGro7 plasmid (*Gro*EL and *Gro*ES) was used as competent cells for transformation. The pET-22b(+) encoding CamA was transformed into the *E. coli* BL21 (DE3) competent cells and the resulting transformants were incubated in LB broth at 37 °C, 220 rpm for 1.5 hr. The cells were then spread on an LB agar with ampicillin (50 µg/ml), and chloramphenicol (20 µg/ml) and incubated at 37 °C for 12 to 16 hr. A single colony was selected for inoculating into a starter culture and 1% (v/v) of the starter was then inoculated into 650 ml of auto-induction medium with antibiotic drugs including ampicillin (50 µg/ml), and chloramphenicol (20 µg/ml). The culture medium was grown at 37 °C, 220 rpm until OD_{600} reached about 0.8-1.0, and then grown at 25 °C for 20 hr before being harvested by centrifugation.

For CamA purification, the cell pellet was resuspended in 50 mM NaH_2PO_4, 300 mM NaCl, and 20 mM imidazole at pH 8.0 and lysed on ice using ultrasonication. After centrifugation, the supernatant was collected, added 0.1% (v/v) polyethylene amine (PEI), centrifuged to discard the pellet and the supernatant was collected for using in further steps. The supernatant was loaded onto a nickel nitriloacetic acid (Ni-NTA) affinity chromatography column. The column was washed and eluted with buffers containing 50 mM and 100 mM imidazole, respectively. The purified enzyme was added flavin adenine dinucleotide (FAD) and then concentrated using a Millipore Ultra-filter unit. The excess FAD was removed at this step. Finally, the enzyme solution was exchanged its buffer into 50 mM NaH_2PO_4, 300 mM NaCl, pH 7.5 using a Sephadex® G-25 gel filtration column.

Expression and purification of putidaredoxin (CamB)

E. coli BL21 (DE3) containing a pGro7 plasmid (*Gro*EL and *Gro*ES) was used as competent cells for transformation. The pET-22b(+) encoding CamB was transformed into the *E. coli* BL21 (DE3) competent cells. The transformants were incubated in LB broth at 37 °C, 220 rpm for 1.5 hr and then spread on an LB agar with ampicillin (50 µg/ml), and chloramphenicol (20 µg/ml) and incubated at 37 °C for 12 to 16 hr. A single colony was selected for inoculating into a starter culture and 1% (v/v) of the starter was then inoculated into 650 ml of auto-induction medium with ammonium iron (III) citrate (20 ug/ml) and antibiotic drugs including ampicillin (50 µg/ml) and chloramphenicol (20 µg/ml). The culture medium was grown at 37 °C, 220 rpm until OD_{600} reached about 0.8-1.0, and then grown at 25 °C for 20 hr before being harvested by centrifugation. The purification method used the same method as CamA without FAD added in the purified enzyme.

Expression and purification of ferredoxin reductase (FdR)

E. coli BL21 (DE3) was used as competent cells for transformation. The RSFDuet-1 encoding FdR was transformed into the *E. coli* BL21 (DE3) competent cells. The transformants were incubated in LB broth at 37 °C, 220 rpm for 1.5 hr. Cells were then spread on an LB agar with kanamycin (34 µg/ml), and incubated at 37 °C for 12 to 16 hr. A single colony was selected for inoculating into a starter culture and 1% (v/v) of the starter was then inoculated into 650 ml of LB medium containing 34 µg/ml kanamycin. The culture medium was grown at 37 °C, 220 rpm until OD_{600} reached about 0.8-1.0. Then, 1 mM of isopropyl β-d-1-thiogalactopyranoside (IPTG) was then added to the culture and grown at 25 °C for 8 hr before being harvested by centrifugation.

For the purification step, the cell pellet was resuspended in the lysis buffer (50 mM NaH_2PO_4, pH 7.0) with 1 mM dithiothreitol (DTT) and 100 µM phenylmethylsulfonyl fluoride (PMSF). The cells were then lysed on ice using ultrasonication. The lysis mixture was then centrifuged to separate supernatant from pellet. After centrifugation, the supernatant was collected and added 0.1% (v/v) PEI. The suspension was centrifuged to discard the pellet and the supernatant was collected for using in further steps. The supernatant was precipitated by 40 -

80% (w/w) of ammonium sulfate and then centrifuged at 4°C for 30 min to collect pellet. The pellet was re-suspended in the lysis buffer and dialysis to remove ammonium sulfate overnight. The crude protein was loaded onto a diethylaminoethyl (DEAE) column that was pre-equilibrated and washed using lysis buffer. The column was then eluted by a gradient of NaCl from 0 to 500 mM in lysis buffer. The yellow fractions were collected, and FAD was added and concentrated using a Millipore Ultra-filter unit. The excess FAD was removed at this step. Finally, the enzyme solution was buffer exchanged into 50 mM NaH_2PO_4, 300 mM NaCl, pH 7.5 using a Sephadex® G-25 gel filtration column. The enzyme solution was stored at − 80 °C until use.

Expression and purification of ferredoxin (FdX)

E. coli BL21 (DE3) was used as competent cells for transformation. The pCDEDuet-1 encoding FdX was transformed into the *E. coli* BL21 (DE3) competent cells. The transformants were incubated in LB broth at 37 °C, 220 rpm for 1.5 hr. Cells were then spread on an LB agar with streptomycin (25 µg/ml) and incubated at 37 °C for 12 to 16 hr. A single colony was selected for inoculating into a starter culture and 1% (v/v) of the starter was then inoculated into 650 ml of LB medium containing streptomycin (25 µg/ml). The culture medium was grown at 37 °C, 220 rpm until OD_{600} reached about 0.8-1.0. Then, 1 mM of IPTG was added and the culture and grown at 25 °C for 8 hr before being harvested by centrifugation.

For purification of FdX, the cell pellet was resuspended in 50 mM NaH_2PO_4, 300 mM NaCl, at pH 7.0, 1 mM DTT, and 100 µM PMSF. Cells were lysed on ice using ultrasonication. After centrifugation, the supernatant was collected and added 0.1% (v/v) PEI. The suspension was then centrifuged to discard the pellet and the supernatant was collected for using in further steps. The supernatant was precipitated by adding 10% (w/w) of ammonium sulfate and then centrifuged at 4°C for 30 min to collect supernatant. The supernatant was loaded onto a Ni-NTA affinity chromatography column. The column was washed and eluted with buffers containing 50 mM and 250 mM imidazole, respectively. Finally, the enzyme solution was exchanged its buffer into 50 mM NaH_2PO_4, 300 mM NaCl, pH 7.5 using a

Sephadex® G-25 gel filtration column. The enzyme solution was stored at − 80 °C until use.

Supplementary Table

Table S1 Yields of purified enzymes and heme content

	**$OleT_{LA}$**	**$OleT_{JE}$**
**P1 method**		
Total purified protein (mg/ L of culture medium)	218.0 ± 0.1	285.0 ± 1.0
Total purified holoenzyme (mg/ L of culture medium)	119.9 ± 0.3	107.40 ± 0.3
Holoenzyme content (%)[a]	55.0 ± 0.1	37.7 ± 0.1
**P2 method**		
Total purified protein (mg/ L of culture medium)	234.7 ± 0.3	233.1 ± 0.2
Total purified holoenzyme (mg/ L of culture medium)	178.1 ± 0.5	171.5 ± 0.8
Holoenzyme content (%)[a]	75.9 ± 0.3	73.1 ± 0.3
**P3 method**		
Total purified protein (mg/ L of culture medium)	226.8 ± 0.1	164.5 ± 0.2
Total purified holoenzyme (mg/ L of culture medium)	169.7 ± 0.7	117.5 ± 0.3
Holoenzyme content (%)[a]	74.8 ± 0.3	71.6 ± 0.2

[a]Holoenzyme content was calculated from percentage of purified holoenzyme per total purified protein

Table S2 Active site cavity of each enzyme

Enzyme	PDB code	Volume (Å^3)	Heme binding mode
**$OleT_{JE}$**	4L40	1149	Usual
**CYP152K6**	6FYJ	1706	Usual
**CYP152A1**	2ZQX	1548	Usual
**CYP152B1**	3AWM	2311	Usual
**CYP152N1**	5YHJ	2637	Unusual
**$OleT_{LA}$**	9JQM	2693	Unusual

Table S3 Genes and plasmids used for overexpression. Antibiotic markers used in this study

Gene	Original organism	Plasmid	Antibiotic resistance gene
**$OleT_{LA}$**	*Lacicoccus alkaliphilus*	pET-22b(+)	Ampicillin
**$OleT_{JE}$**	*Jeotgalicoccus* sp. ATCC 8456	pET-22b(+)	Ampicillin
***Gro*EL-*Gro*ES**	-	pGro7	Chloramphenicol

**Outer membrane heme receptor (ChuA)**	*Escherichia coli* Nissle 1917	pRSFDuet-1	Kanamycin
**Ferrochelatase (FC)**	*Escherichia coli*	pRSFDuet-1	Kanamycin
**Aminolevulinic acid synthase (ALAS)**	*Rhodobacter capsulatus*	pRSFDuet-1	Kanamycin
**FC-ALAS**	*Escherichia coli* (FC) and *Rhodobacter capsulatus* (ALAS)	pRSFDuet-1	Kanamycin
**FdX**	*Synechocystis* sp. PCC 6803	pCDFDuet-1	Streptomycin
**FdR**	*Escherichia coli* K-12	pRSFDuet-1	Kanamycin
**CamA**	*Pseudomonas putida*	pET-22b(+)	Ampicillin
**CamB**	*Pseudomonas putida*	pET-22b(+)	Ampicillin
**FdX-FdR**	*Synechocystis* sp. PCC 6803 (FdX) and *Escherichia coli* K-12 (FdR)	pRSFDuet-1	Kanamycin

Table S4 List of primers for site-directed mutagenesis

Primer	Sequence 5' → 3'
**Forward OleT$_{LA}$ I178L**	CAGCTTCAAAGCGCTGGGCAGCGCGTTTAAGGG
**Reverse OleT$_{LA}$ I178L**	CTTAAACGCGCTGCCCAGCGCTTTGAAGCTGTC
**Forward OleT$_{JE}$ L176I**	TTCCGTGCGATTGGTGGCGCGTTTAAGGG
**Reverse OleT$_{JE}$ L176I**	AAACGCGCCACCAATCGCACGGAAGCTGTC

Supplementary figures

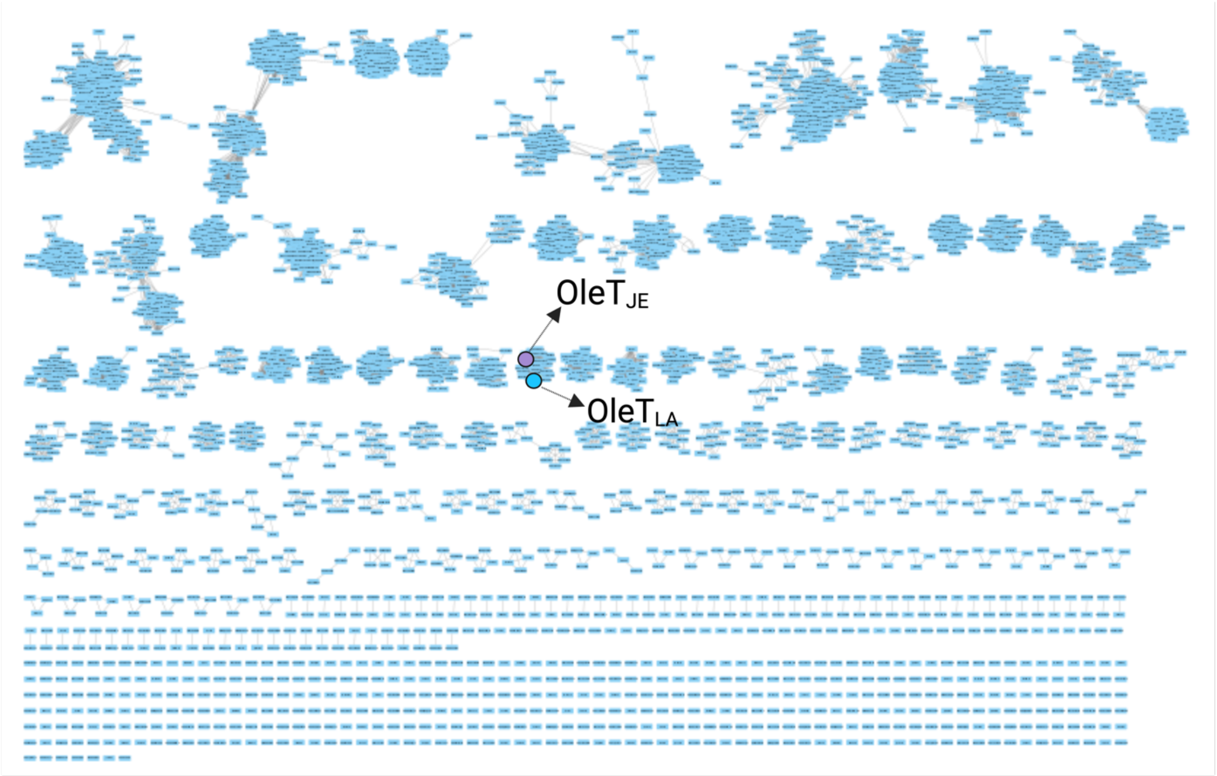

Figure S1. Identification of OleT$_{LA}$ from a sequence similarity network (SSN) of OleT$_{JE}$. Sequence similarity networks of CYP152 enzymes with sequence identity higher than 60% to OleT$_{JE}$ were constructed by the Enzyme Function Initiative-Enzyme Similarity Tool (EFI-EST) tool. The OleT$_{LA}$ and OleT$_{JE}$ are located in the same cluster and indicated as blue and purple circles, respectively.

	CYP152T7	OleTPRN	CYP152T8	CYP152T1	OleTJE	CYP152JH	CYP152L8	OleTLA	CYP152L2	CYP152L7	CYP152MC	CYP152MP	CYP152B1	P450Jα	CYP152N1	CYP152K6	CYP152A2	CYP152A1	CYP152A8
CYP152T7	100.0	49.3	54.5	56.8	28.3	28.5	29.1	29.6	27.7	27.4	27.4	30.2	32.7	31.1	29.4	28.0	28.5	32.5	30.6
OleTPRN	49.3	100.0	52.9	55.3	31.1	31.1	33.4	32.9	28.3	30.2	29.0	27.7	32.1	31.2	30.5	29.7	31.4	31.2	31.7
CYP152T8	54.5	52.9	100.0	62.5	28.4	27.6	30.1	28.0	27.8	27.5	28.5	32.6	32.5	29.2	30.4	28.2	32.1	32.5	31.3
CYP152T1	56.8	55.3	62.5	100.0	29.2	28.1	29.5	29.8	26.3	27.7	25.6	29.5	33.3	29.8	28.8	29.8	30.0	34.2	31.5
OleTJE	28.3	31.1	28.4	29.2	100.0	92.4	76.3	74.6	63.5	64.2	59.0	31.1	36.2	37.2	34.6	37.6	38.9	41.0	39.2
CYP152JH	28.5	31.1	27.6	28.1	92.4	100.0	76.8	75.1	62.4	64.3	59.3	31.1	35.8	36.6	35.2	37.5	37.6	39.7	38.7
CYP152L8	29.1	33.4	30.1	29.5	76.3	76.8	100.0	91.0	62.8	67.1	63.0	30.7	36.0	37.0	37.0	40.2	40.6	41.5	39.5
OleTLA	29.6	32.9	28.0	29.8	74.6	75.1	91.0	100.0	62.8	66.1	62.6	32.1	36.0	37.0	36.3	41.0	40.8	40.5	39.0
CYP152L2	27.7	28.3	27.8	26.3	63.5	62.4	62.8	62.8	100.0	69.6	63.7	29.5	35.4	35.8	37.8	39.1	38.0	36.9	37.4
CYP152L7	27.4	30.2	27.5	27.7	64.2	64.3	67.1	66.1	69.6	100.0	68.9	31.7	34.0	35.1	40.0	41.7	38.2	38.9	38.7
CYP152MC	27.4	29.0	28.5	25.6	59.0	59.3	63.0	62.6	63.7	68.9	100.0	30.5	32.8	38.0	40.2	39.6	38.2	38.4	39.0
CYP152MP	30.2	27.7	32.6	29.5	31.1	31.1	30.7	32.1	29.5	31.7	30.5	100.0	43.6	35.1	38.4	37.6	40.1	44.6	45.5
CYP152B1	32.7	32.1	32.5	33.3	36.2	35.8	36.0	36.0	35.4	34.0	32.8	43.6	100.0	39.4	44.0	41.9	42.5	43.9	42.7
P450Jα	31.1	31.2	29.2	29.8	37.2	36.6	37.0	37.0	35.8	35.1	38.0	35.1	39.4	100.0	47.3	46.4	41.9	44.2	43.8
CYP152N1	29.4	30.5	30.4	28.8	34.6	35.2	37.0	36.3	37.8	40.0	40.2	38.4	44.0	47.3	100.0	48.9	42.7	43.8	45.0
CYP152K6	28.0	29.7	28.2	29.8	37.6	37.5	40.2	41.0	39.1	41.7	39.6	37.6	41.9	46.4	48.9	100.0	48.8	49.6	49.2
CYP152A2	28.5	31.4	32.1	30.0	38.9	37.6	40.6	40.8	38.0	38.2	38.2	40.1	42.5	41.9	42.7	48.8	100.0	59.1	57.9
CYP152A1	32.5	31.2	32.5	34.2	41.0	39.7	41.5	40.5	36.9	38.9	38.4	44.6	43.9	44.2	43.8	49.6	59.1	100.0	64.3
CYP152A8	30.6	31.7	31.3	31.5	39.2	38.7	39.5	39.0	37.4	38.7	39.0	45.5	42.7	43.8	45.0	49.2	57.9	64.3	100.0

Figure S2. Sequence identity matrix of $OleT_{LA}$ and the previously reported CYP152 enzymes. The green-to-red color represents high-to-low sequence identity.

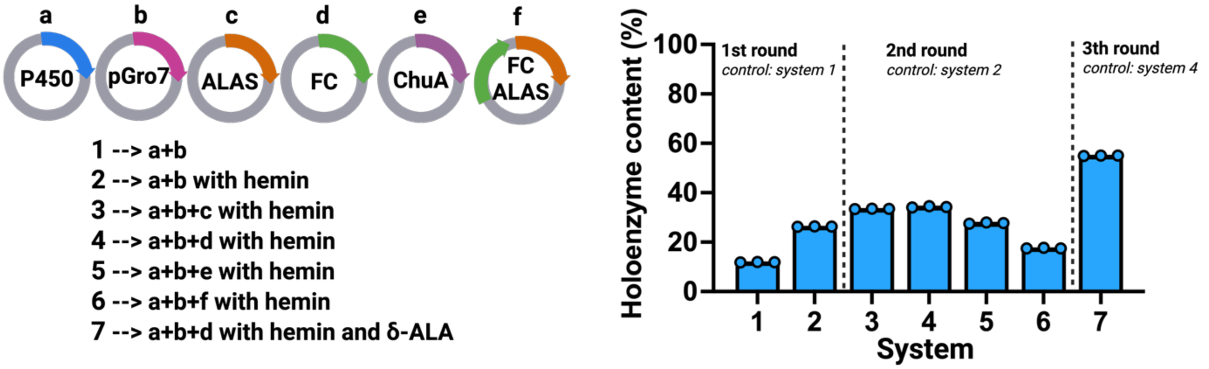

Figure S3. Improvement of overexpression of CYP152 holoenzymes. Experimental design to improve production of the holoenzyme of $OleT_{LA}$ using Systems 1-7 which co-expressed additional genes encoding for proteins related to heme synthesis. The plasmids used included : a, CYP152 gene in the pET-22b(+) vector; b, *Gro*EL and *Gro*ES in the pGro7 vector; c, d, e, f are genes encoding ALA synthase (ALAS), ferrochelatase (FC), outer membrane heme receptor (ChuA), and FC and ALAS, respectively, in the pRSFDuet-1 vector. The experiments were conducted in multiple rounds: **First round:** We compared System 1 and System 2 and found that the presence of hemin in System 2 resulted in higher heme content. Therefore, System 2 was used as a control in the second round. **Second round:** We compared Systems 2, 3, 4, 5, and 6. System 2 served as a control. All systems included hemin in the culture media. The results showed that System 4 produced the highest heme content, indicating that expression of the ferrochelatase gene can enhance holoenzyme expression. Based on these data, System 4 was selected as a control in

the third round. **Third round:** We tested effects of δ-ALA supplementation on System 4 by adding δ-ALA to the culture medium (System 7). The results demonstrated an increase in the heme content in System 7. Altogether, the data support that System 7 is the best one to give the highest holoenzyme expression and all components in System 7 (combination of hemin and δ-ALA supplementation and co-expression with ferrochelatase) are necessary to enhance holoenzyme expression of CYP152.

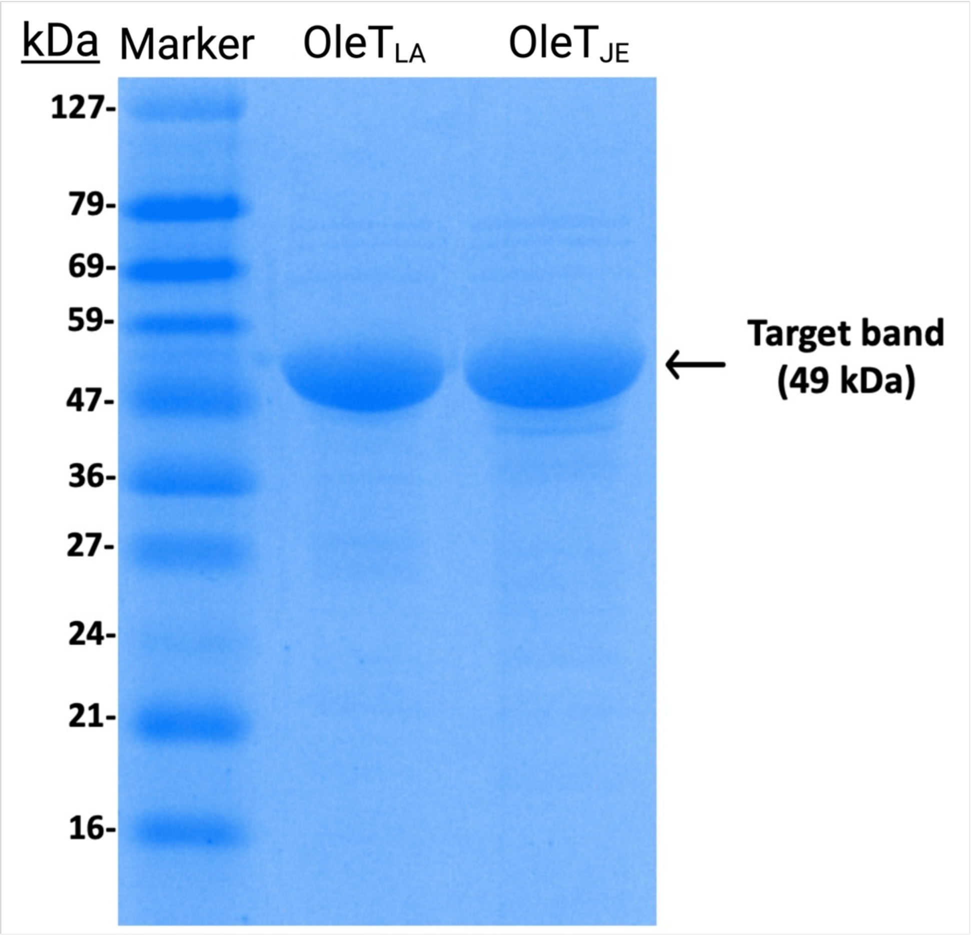

Figure S4. SDS-PAGE of purified OleT$_{LA,}$ and OleT$_{JE.}$ Both enzymes showed the correct band at 49 kDa.

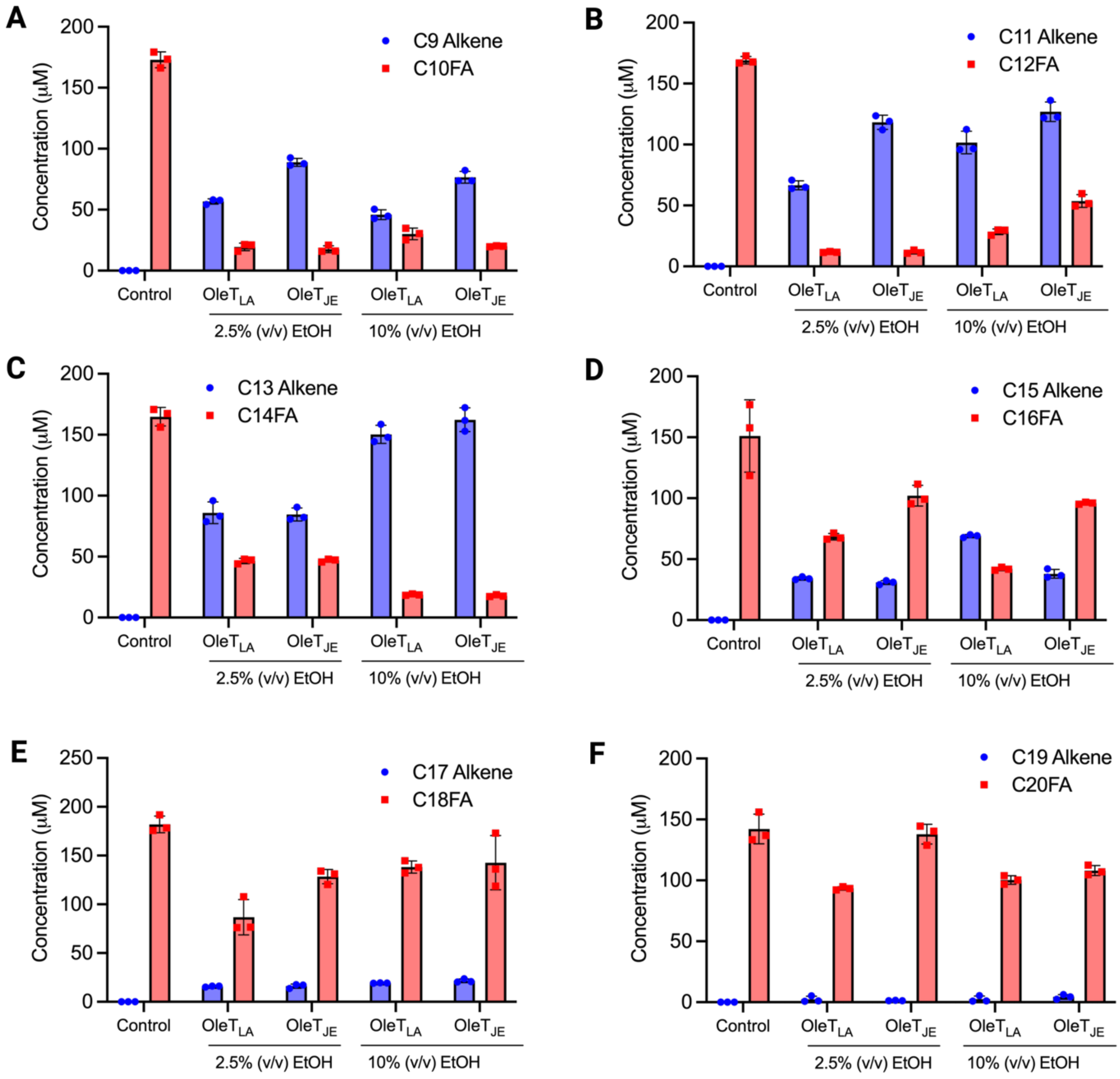

Figure S5. Alkene production yields and remaining fatty acid in the reaction of the OleT$_{LA}$ and OleT$_{JE}$ reactions using fatty acids with various chain lengths. *A*, C10FA; *B*, C12FA; *C*, C14FA; *D*, C16FA; *E*, C18FA; and *F*, C20FA in 2.5% and 10% (v/v) EtOH as co-solvent. All reactions contained 5 µM enzyme, 200 µM of substrate, and 400 µM of H_2O_2. The control reaction was performed in the absence of enzyme in 2.5% (v/v) EtOH as co-solvent. The reactions were performed at 25 °C for 1 hr and then quenched by adding 2-fold volume of ethyl acetate. Alkene product analysis was done by GC-MS.

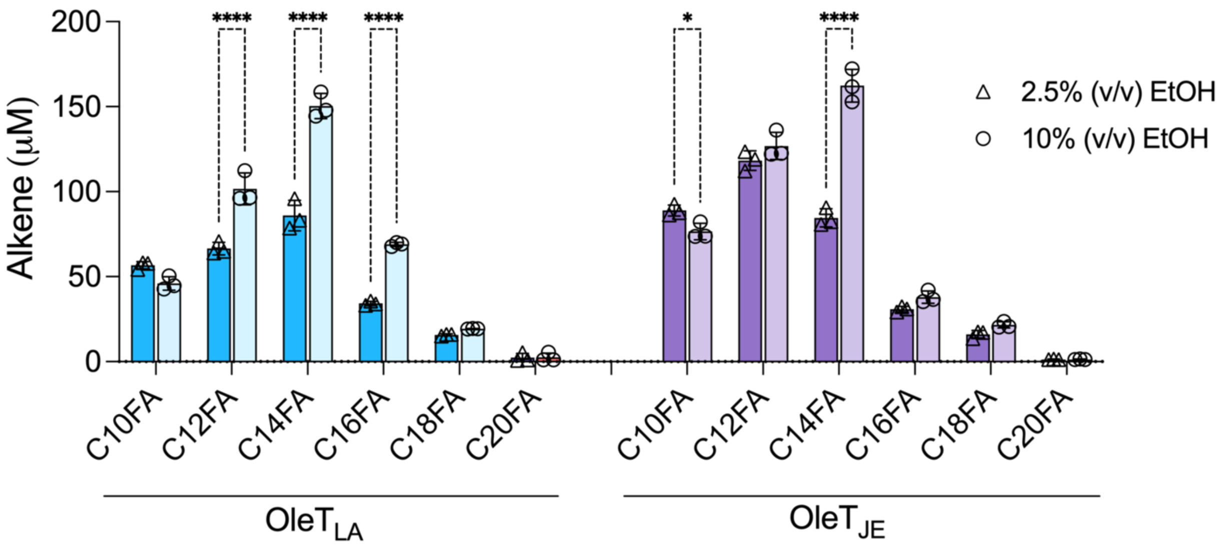

Figure S6. Alkene production yields from the reactions of OleT$_{LA}$ and OleT$_{JE}$. The reactions of OleT$_{LA}$ and OleT$_{JE}$ in 2.5% (v/v) EtOH as co-solvent are shown in dark blue and dark purple, respectively. The reactions of OleT$_{LA}$ and OleT$_{JE}$ in 10% (v/v) EtOH as co-solvent are shown in light blue and light purple, respectively. All reactions contained 5 µM of enzyme, 200 µM of substrate, and 400 µM of H_2O_2. The reactions were performed at 25 °C for 1 hr and then quenched by adding 2-fold volume of ethyl acetate. Alkene product analysis was done by GC-MS. Results are shown as the mean ± SD (n = 3). Asterisks denote statistical significances (****$p \leq 0.001$; *$p \leq 0.05$) as determined by two-way ANOVA followed by multiple comparison tests.

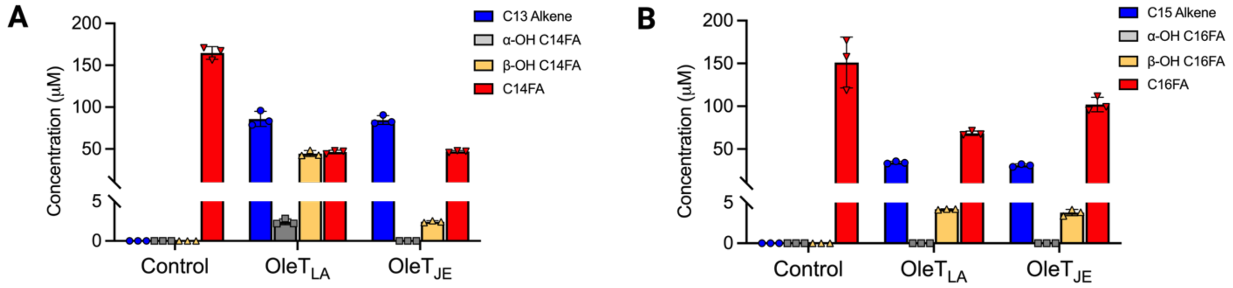

Figure S7. Distribution of products from the reactions of $OleT_{LA}$ and $OleT_{JE}$ which used *A,* C14FA, and *B,* C16FA as substrates. The reactions contained 5 μM enzyme, 200 μM substrate, and 400 μM H_2O_2 and were carried out at 25 °C for 1 hr. The single hydroxylated products of C14FA and C16FA were detected by negative SIM mode with m/z 243 and 271, respectively.

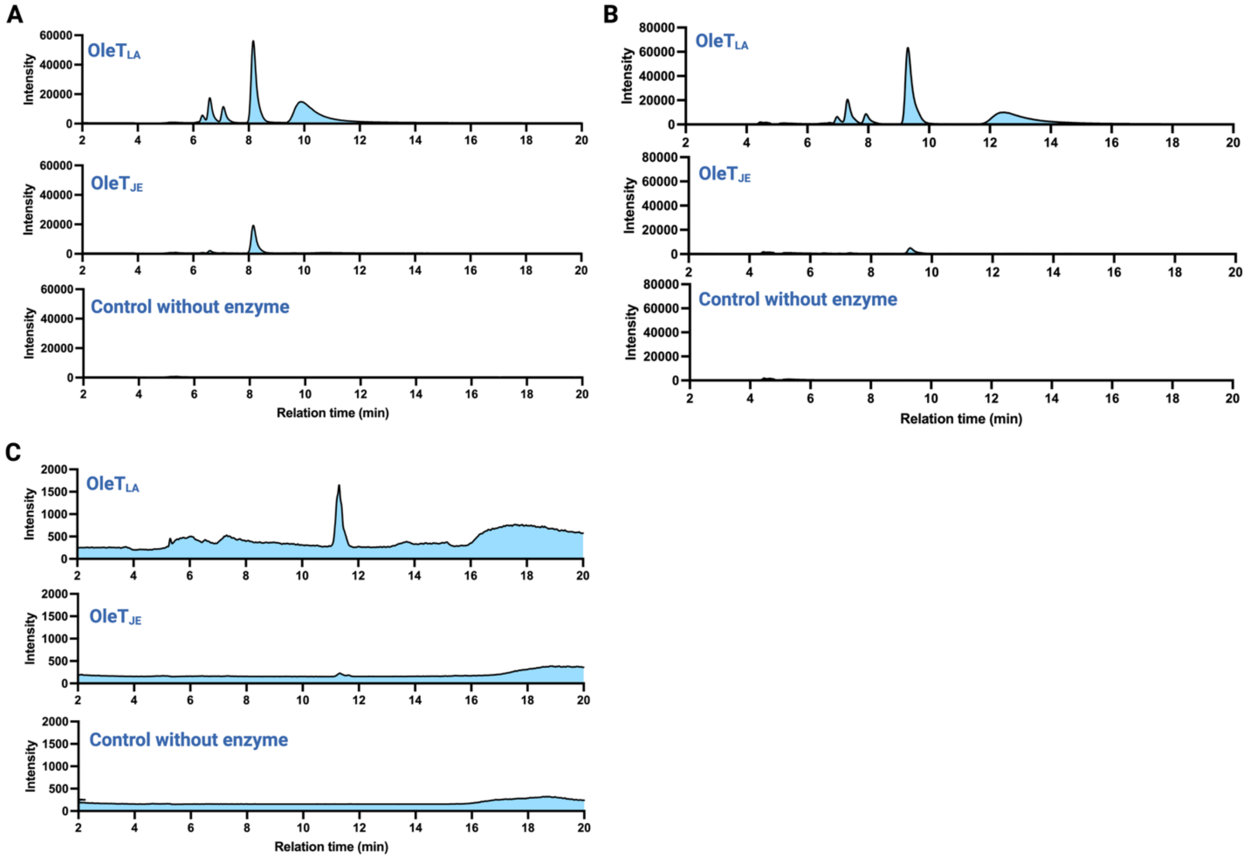

Figure S8. Chromatograms of single hydroxylated products of OleTLA and OleTJE. *A,* Hydroxylated products detected in negative SIM mode (m/z 187) using C10FA as the substrate. *B,* Hydroxylated products detected in negative SIM mode (m/z 215) using C12FA as the substrate. *C*, Hydroxylated products detected in negative SIM mode (m/z 299) using C18FA as the substrate.

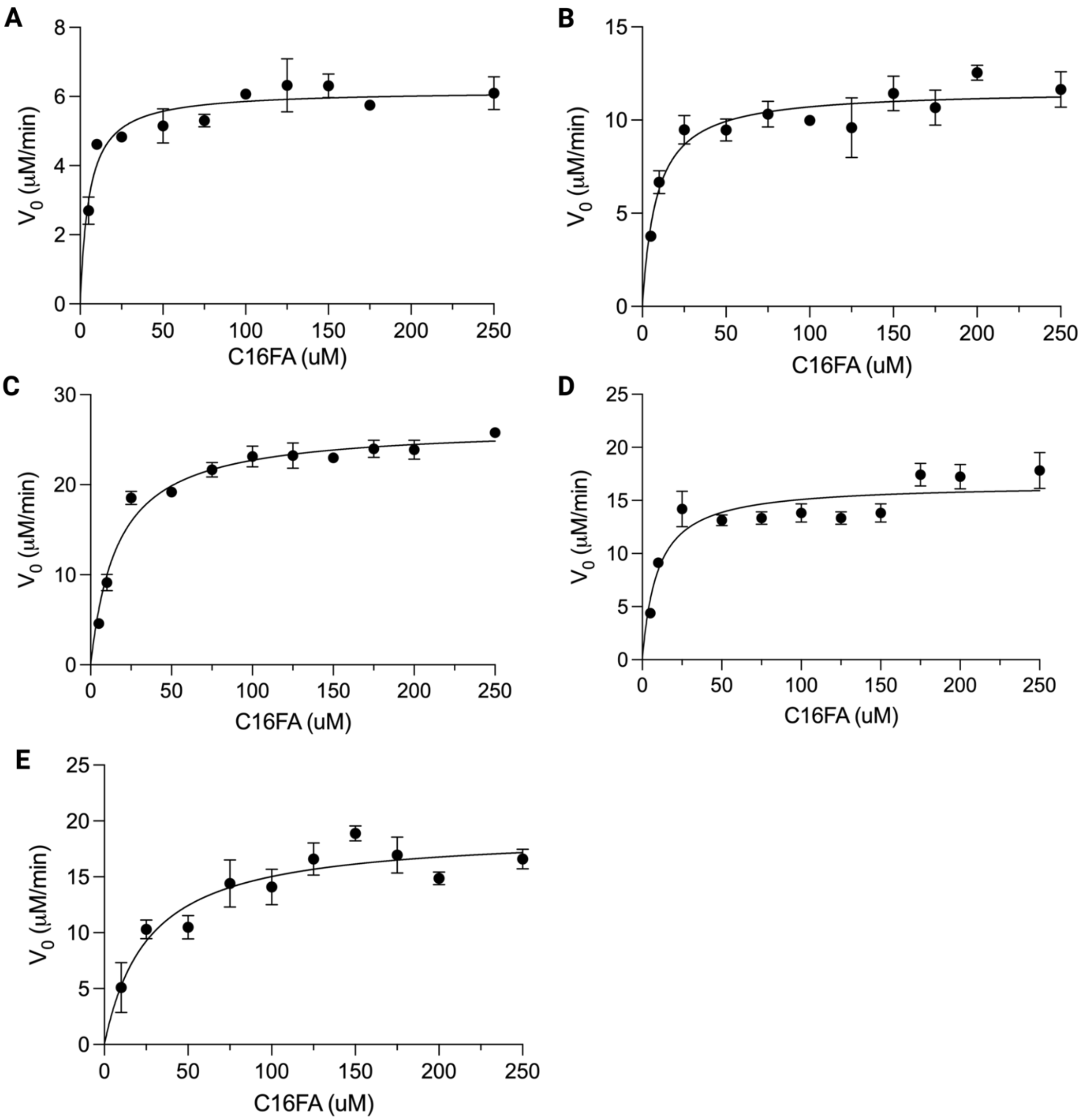

Figure S9. Steady-state kinetics of the reactions of $OleT_{LA}$ and $OleT_{JE}$ under various conditions. *A*, Reactions contained $OleT_{LA}$ (0.3 μM), H_2O_2 (400 μM) and C16FA (5-250 μM) in 50 mM NaH_2PO_4, 300 mM NaCl pH 7.5, 2.5% (v/v) EtOH. *B*, Reactions contained $OleT_{JE}$ (0.3 μM), H_2O_2 (400 μM) and C16FA (5-250 μM) in 50 mM NaH_2PO_4, 300 mM NaCl pH 7.5, 2.5% (v/v) EtOH. *C*, Reactions contained $OleT_{LA}$ (0.3 μM), H_2O_2 (400 μM) and C16FA (5-250 μM) in 50 mM NaH_2PO_4, 300 mM NaCl pH 7.5, 10% (v/v) EtOH. *D*, Reactions contained $OleT_{JE}$ (0.3 μM), H_2O_2 (400 μM) and C16FA (5-250 μM) in 50 mM NaH_2PO_4, 300 mM NaCl pH 7.5, 10% (v/v) EtOH. *E*,

OleT$_{LA}$ I178L (0.3 µM), H_2O_2 (400 µM) in 50 mM NaH_2PO_4, 300 mM NaCl pH 7.5 containing 10% (v/v) EtOH.

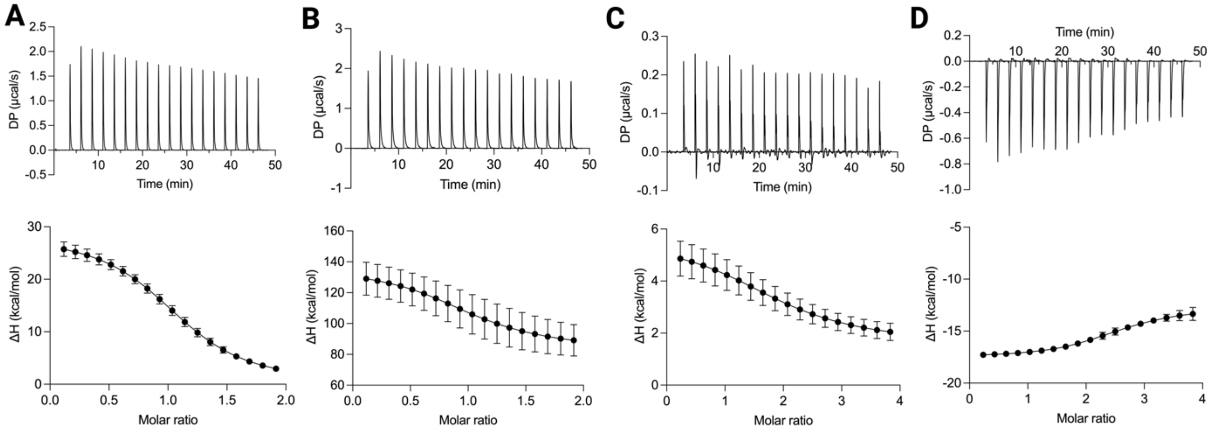

Figure S10. Calorimetric titrations (upper panel) and integrated binding isotherms (lower panel) for bindings of OleT$_{LA}$ and OleT$_{JE}$ with C16FA under various conditions. *A*, OleT$_{LA}$ in 2.5% (v/v) EtOH; *B*, OleT$_{LA}$ in 10% (v/v) EtOH; *C,* OleT$_{JE}$ in 2.5% (v/v) EtOH; *D*, OleT$_{JE}$ in 10% (v/v) EtOH with C16FA. The integrated binding isotherms were plotted using data from three experimental replicates.

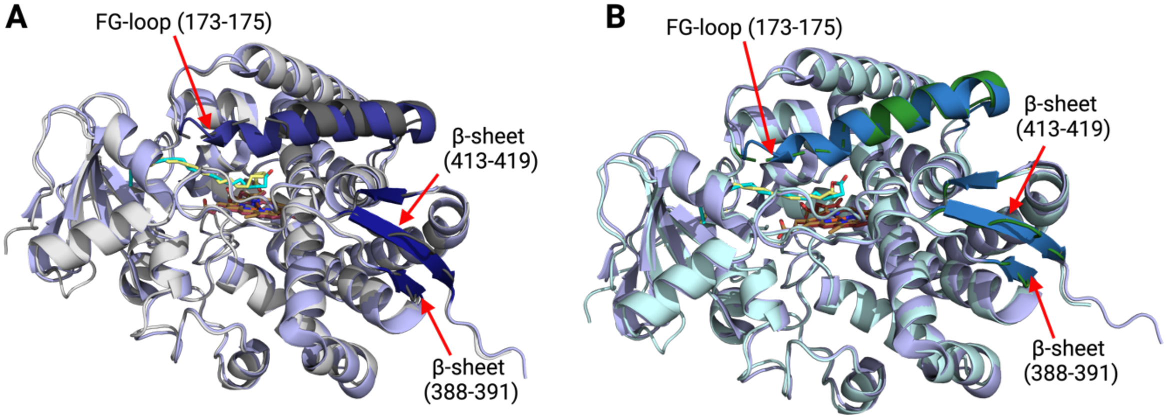

Figure S11. Comparison of the overall structures and heme-binding sites of OleT$_{LA}$ and OleT$_{JE}$. *A*, Alignment of Chain A of OleT$_{LA}$: C20FA (PDB: 9JQM) and OleT$_{JE}$: C20FA (PDB: 4L40) structures. The significant differences between Chain A of OleT$_{LA}$ and OleT$_{JE}$ are represented as *black* and *blue*, respectively. *B*, Alignment of Chain B of OleT$_{LA}$: C20FA and OleT$_{JE}$: C20FA structures. The significant differences between Chain B of OleT$_{LA}$ and OleT$_{JE}$ are represented as *green* and *blue*, respectively.

```
OleTJE      ---MATLKRDKGLDNTLKVLKQGYLYTTNQRNRLN--TSVFQTKALGGKPFVVVTGKEGA   55
CYP152A1    --MNEQIPHDKSLDNSLTLLKEGYLFIKNRTERYN--SDLFQARLLG-KNFICMTGAEAA   55
CYP152B1    ---MPKTPHTKGPDETLSLLADPYRFISRQCQRLG--ANAFESRFLL-KKTNCLKGAKAA   54
CYP152K6    MSNINQMPREEGIDSTWRLMEEGYMYILNRRHSFN--SDIFETRLLG-KKAICMGGKEAA   57
CYP152N1    --MGKVIPKQEGLDHSVDFLREGYLFVANRRKSFQ--SNIFESRLLG-ERVICLGGEEAA   55
OleTLA      ---MATIKKDKGIDNTAKIAKQGYLYTTNQRERLGVKDGVFETRGLGGKRIIILSGKDGA   57

OleTJE      EMFYNNDVVQREGMLPKRIVNTLFGKGAIHTVDGKKHVDRKALFMSLMTEGNLNYVRELT   115
CYP152A1    KVFYDTDRFQRQNALPKRVQKSLFGVNAIQGMDGSAHIHRKMLFLSLMTPPHQKRLAELM   115
CYP152B1    EIFYDTTRFEREGAMPVAIQKTLLGQGGVQGLDGETHRHRKQMFMGLMTPERVRALAQLF   114
CYP152K6    EIFYDTEKFKRKDAAPNRVVQTLFGKNGVQALDGQTHKHRKEMFMSIMSPDELEKLTDIT   117
CYP152N1    EVFYDANKFTRQDAAPKRLLKTLFGEGGVQTLDGSEHTHRKQMFMSLMTKENIDRLLRLT   115
OleTLA      ELFYDNDKVERSGTLPKRVVNTLFGKGAIHTTTGKVHVDRKALFMSLMTEGNLQYLRELT   117

OleTJE      RTLWHANTQRMESMDEVNIYRESIVLLTKVGTRWAGVQAPPEDIERIATDMDIMIDSFRA   175
CYP152A1    TEEWKAAVTRWEKADEVVLFEEAKEILCRVACYWAGVPLKETEVKERADDFIDMVDAFGA   175
CYP152B1    EAEWRRAVPGWTRKGEIVFYDELHEPLTRAVCAWAGVPLPDDEAGNRAGELRALFDAAGS   174
CYP152K6    KKQWEIAVDKWEQMDKVILYEEAKEIMCRTACQWAGVPVQENEVKRLTKNLGAMFESAAA   177
CYP152N1    YREWNQIER---MGEEIVLYDIAQEVLMKAVCEWSGVPLAKEEVGKRTEEMRLLFESGTS   172
OleTLA      RNHWLMNTQRMENMDQVNVYRESIILLTKVGTRWAGVQAPEKEIENIATDMDIMIDSFKA   177

OleTJE      LGGAFKGYKASKEARRRVEDWLEEQIIETRKGNIHPPEGTALYEFAHWEDYLGNPMDSRT   235
CYP152A1    VGPR---HWKGRRARPRAEEWIEVMIEDARAGLLKTTSGTALHEMAFHTQEDGSQLDSRM   232
CYP152B1    ASPR---HLWSRLARRRVDAWAKRIIEGIRAGSIGSGSGTAAYAIAWHRDRHDDLLSPHV   231
CYP152K6    VGLK---HWLGRHARNYEEIWIEELIDRVRDGKVNPPENTTLHKFSWYRDLEGNLLDTET   234
CYP152N1    LGPT---YLQGRKARSSAEVWIRQMVKEVRSNRLLPNEHTALYEFSWHRDESGELLPEEV   229
OleTLA      IGSAFKGYRASKAARRRVEDWLEDQIIQTRKGKIHPPKGTALYEFAHWKDYKGEPMDSRL   237

OleTJE      CAIDLMNTFRPLIAINRFVSFGLHAMNENPITREKIKS-EPDYAYKFAQEVRRYYPFVPF   294
CYP152A1    AAIELINVLRPIVAISYFLVFSALALHEHPKYKEWLRSGNSREREMFVQEVRRYYPFGPF   292
CYP152B1    AAVELVNVLRPTVAIAVYITFVAHALQTCSGIRAALVQ-QPDYAELFVQEVRRFYPFFPA   290
CYP152K6    AAVEVINILRPIVAIAIFINFIALALHHYPEEKEKLKSGDKKYSQMFVQEVRRFYPFFPF   294
CYP152N1    VAVEVLNILRPTVAISVYVLFTVLALHQFPDVKEQVERGEVS-KTEFVQEVRRFYPFFPV   288
OleTLA      CGIDLMNTFRPLIAINRFIAFGALAMHENPVAREKIKQ-DDDYAYMFAQEVRRFYPFVPY   296

OleTJE      LPGKAKVDIDFQGVTIPAGVGLALDVYGTTHDESLWDDPNEFRPERFETWDGSPFDLIPQ   354
CYP152A1    LGALVKKDFVWNNCEFKKGTSVLLDLYGTNHDPRLWDHPDEFRPERFAEREENLFDMIPQ   352
CYP152B1    VVARASQDFEWEGMAFPEGRQVVLDLYGSNHDAATWADPQEFRPERFRAWDEDSFNFIPQ   350
CYP152K6    VVALVKKDFTWKGYKFEEGTLTLLDLYGTNHDPEIWKNPDVFSPDRFAKWEGSPFSFIPQ   354
CYP152N1    AAARVKTDFEWDGYAFPEGTLTLLDLYGTNHDVSIWTEPDRFDPSRFKDWKESPFNFIPQ   348
OleTLA      LPGKVKEDFQYKGYDFEKDTMLAIDIYGTMHDPDVWENPNEFYPERFKDWDGSPFDLIPQ   356

OleTJE      GGGDYWTNHRCAGEWITVIIMEETMKYFAEKITYDVPEQDLEVDLNSIPGYVKSGFVIKN   414
CYP152A1    GGGHAEKGHRCPGEGITIEVMKASLDFLVHQIEYDVPEQSLHYSLARMPSLPESGFVMSG   412
CYP152B1    GGGDHYLGHRCPGEWIVLAIMKVAAHLLVNAMRYDVPDQDLSIDFARLPALPKSGFVMRN   410
CYP152K6    GGGDYFMGHRCAGEWVTIEVMKVSLDYLTNRMDYEVPDQDLSFSMASMPSIPHSKVVIKN   414
CYP152N1    GGGDVDFGHRCAGEHVTIAILAQVIELFTKEYAYTVPPQDLSYSFVDMPSLPKSKLRLTH   408
OleTLA      GGGDYYTNHRCAGEWMTIIIMQETMKYFASRITYDVPEQDLTVDLNSLPGYVKSGFIIEN   416

OleTJE      VREVVDRT   422
CYP152A1    IRRKS---   417
CYP152B1    VHIGG---   415
CYP152K6    VKKRI---   419
CYP152N1    LTRNQ---   413
OleTLA      VREVVDRD   424
```

Figure S12. The multiple sequence alignment of enzymes that have reported structure. The black label is CYP152s with usual heme binding, and the red label is unusual heme binding. The yellow highlight indicates residues around the heme binding site.

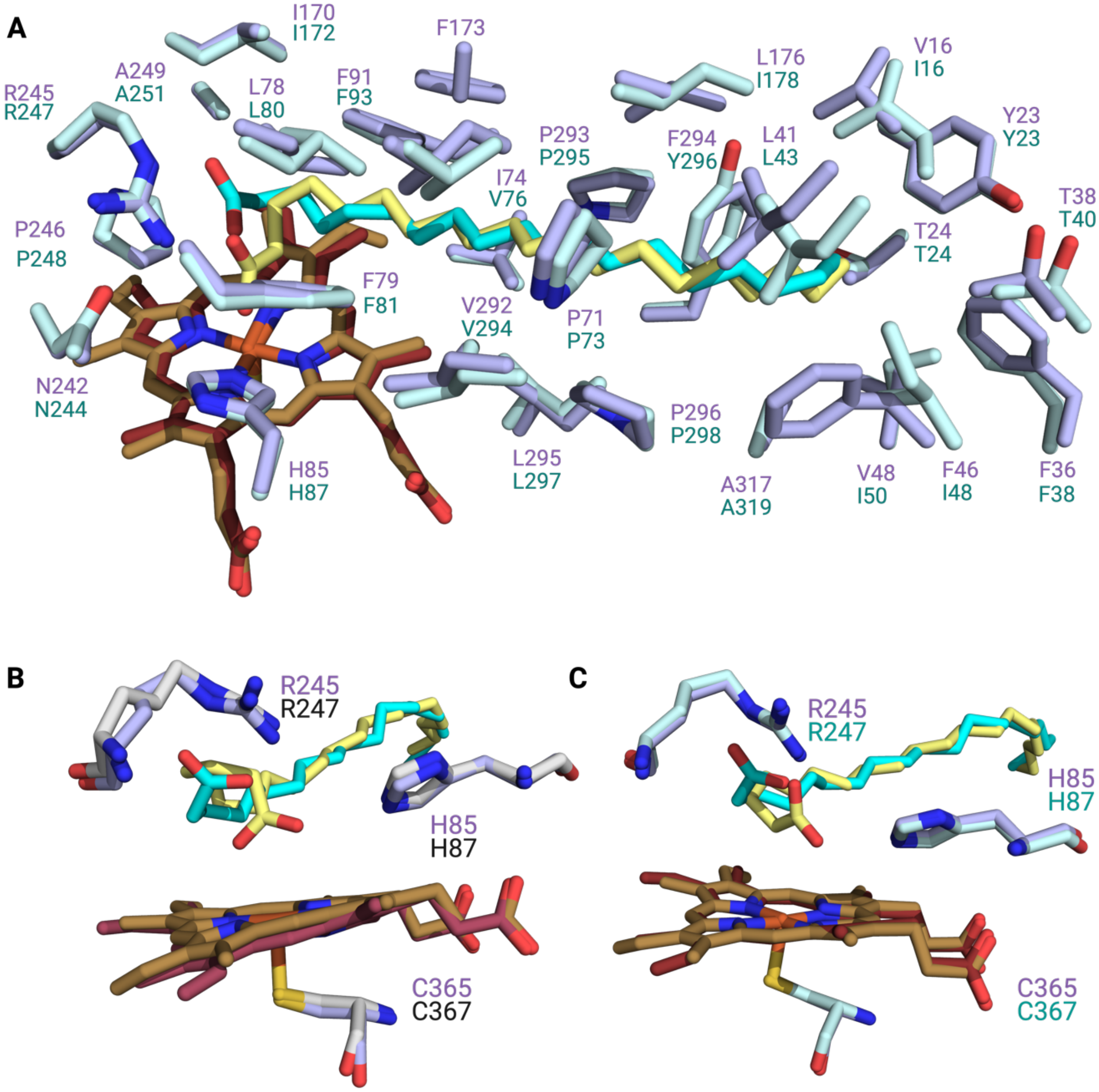

Figure S13. Comparing substrate binding sites of OleT$_{LA}$ and OleT$_{JE}$. *A,* Overlaid substrate binding sites of OleT$_{LA}$: C20FA (PDB: 9JQM) and OleT$_{JE}$: C20FA (PDB: 4L40). In the OleT$_{LA}$ structure, C20FA is depicted as *yellow sticks*, alongside *pale cyan sticks* representing the protein backbone and *brown sticks* for the heme group. In the OleT$_{JE}$ structure, C20FA is illustrated as *cyan sticks*, while *purple sticks* depict the protein backbone, and *red sticks* represent the heme group. *B,* Key catalytic residues at the heme-iron center of Chain A of OleT$_{LA}$ (*gray sticks*) compared to

OleT$_{JE}$ (*purple sticks*). *C*, Key catalytic residues at the heme-iron center of Chain B of OleT$_{LA}$ (*pale cyan sticks*) compared to OleT$_{JE}$ (*purple sticks*).

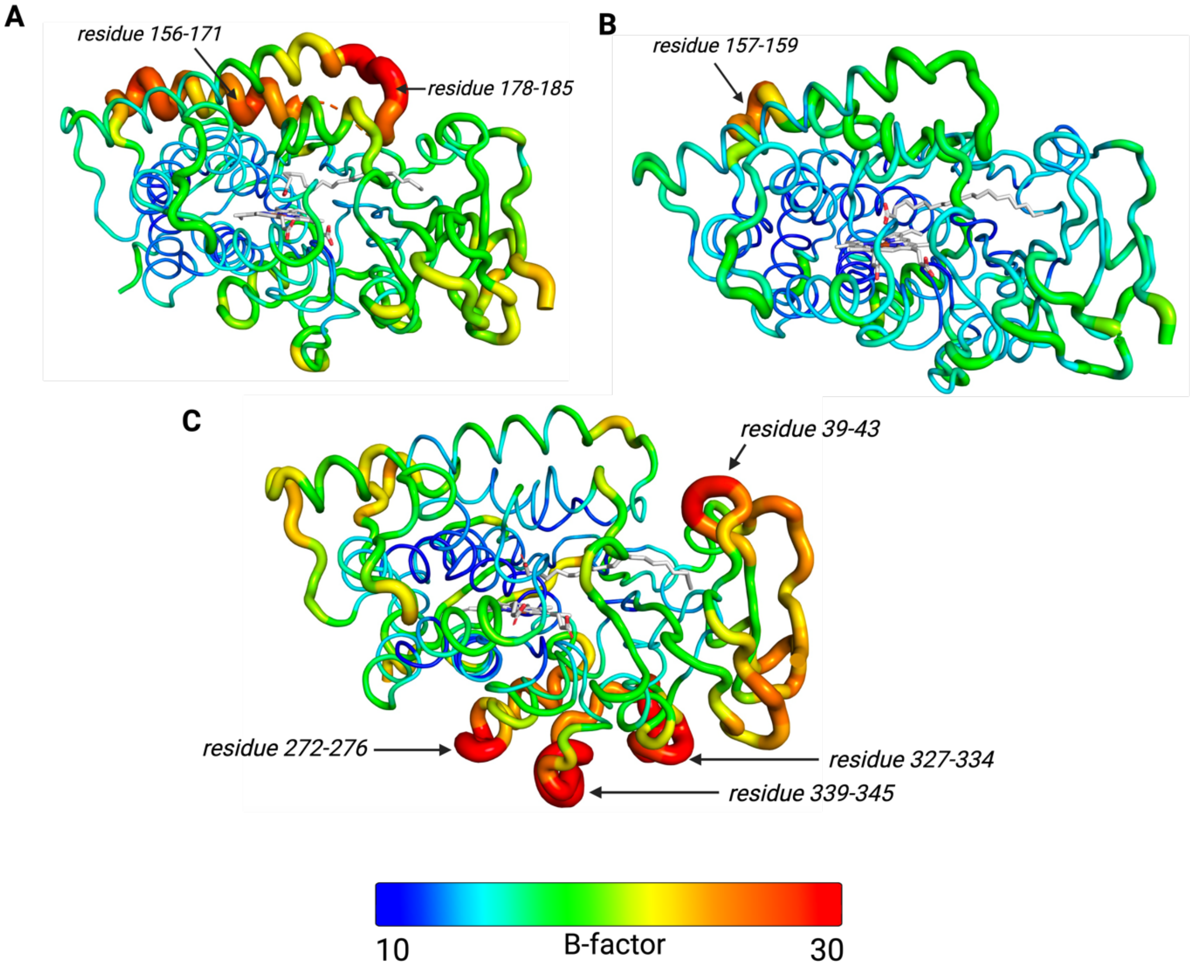

Figure S14. Analysis of flexible regions using PyMOL with preset B-factor. High B-factors are represented by red and yellow regions in the structure of *A*, chain A of OleT$_{LA}$; *B,* chain B of OleT$_{LA}$, and *C,* OleT$_{JE}$.

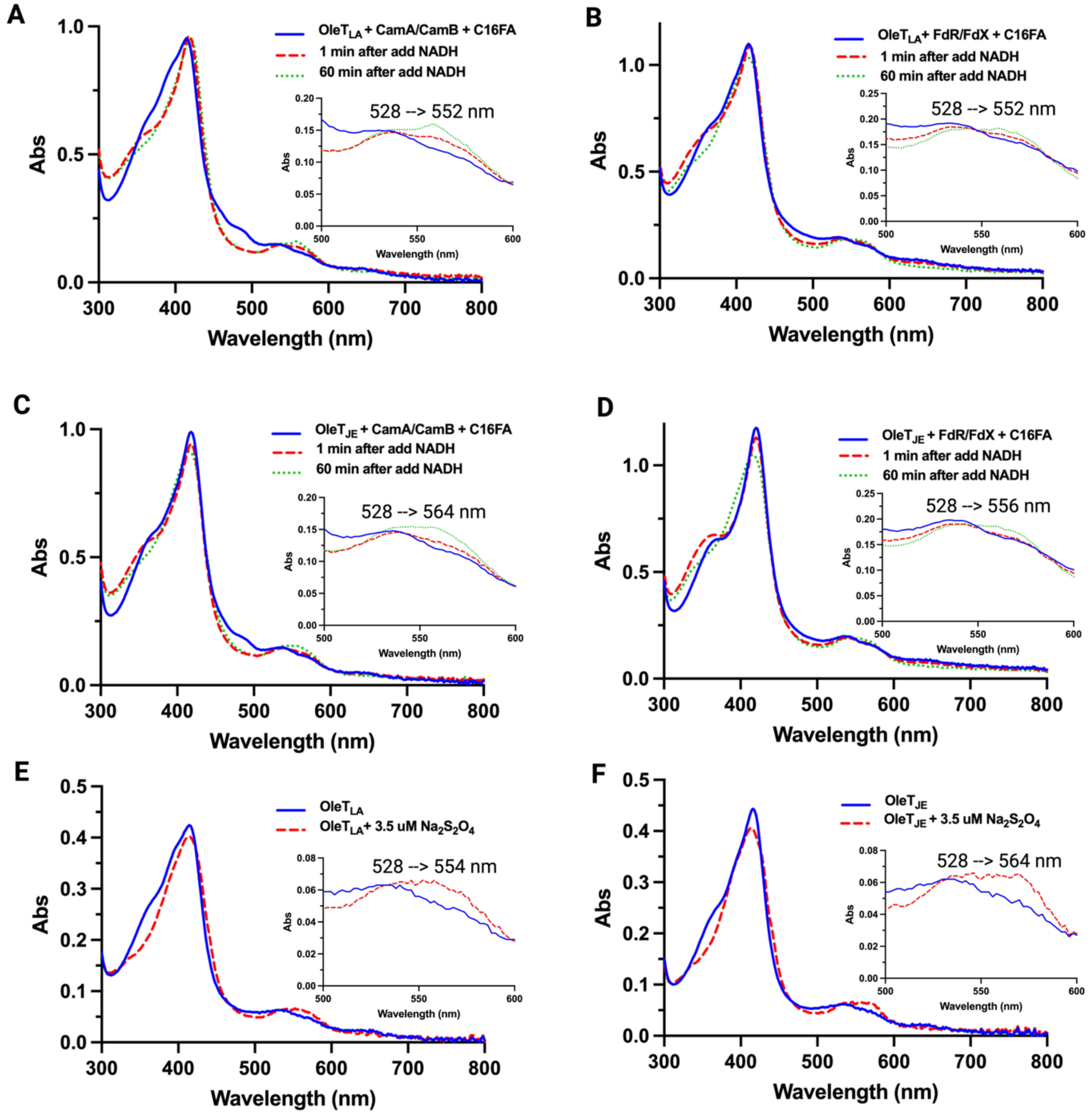

Figure S15. Spectra of ferric and ferrous states of $OleT_{LA}$ and $OleT_{JE}$. *A* and *B* are spectra of $OleT_{LA}$ reduced by CamA/CamB and FdR/FdX, respectively. *C* and *D* are spectra of $OleT_{JE}$ reduced by CamA/CamB and FdR/FdX, respectively. *E* and F are spectra of $OleT_{LA}$ and $OleT_{JE}$, that were reduced by sodium dithionite, respectively. For *A-D*, the spectra of $OleT_{LA}$ or $OleT_{JE}$ incubated with FdR/FdX or CamA/CamB and C16FA are shown in solid blue lines. Spectra after adding NADH for 1 and 60 min are shown as red dashed and green dotted lines, respectively. For *E-F*, the spectra of $OleT_{LA}$ or $OleT_{JE}$ incubated with C16FA are shown in solid blue lines while spectra after adding sodium dithionite for 1 min are shown in dashed red lines. All reactions

were performed in an anaerobic glovebox. The insets display the shift of enzyme spectra from the ferric state (528 nm) to the ferrous state (around 552 for $OleT_{LA}$ and around 560 for $OleT_{JE}$).

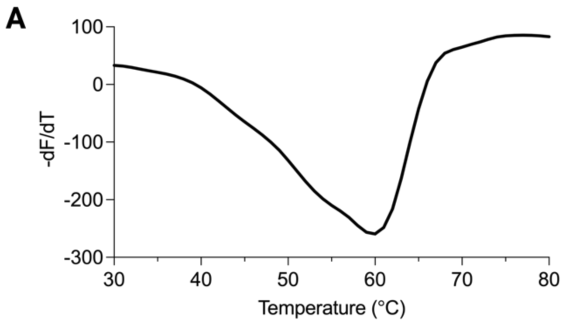

Figure S16. Measurement of melting temperatures of *A,* FdR and *B,* CamA using thermofluor assays.

References

1. Barr, I., and Guo, F. (2015) Pyridine hemochromagen assay for determining the concentration of heme in purified protein solutions. *Bio Protoc*. **5**, e1594
